# Health Impacts of the Built and Social Environments, and Travel Behavior: The Case of the Sunshine State

**DOI:** 10.3390/ijerph19159102

**Published:** 2022-07-26

**Authors:** Jina Mahmoudi, Lei Zhang

**Affiliations:** Maryland Transportation Institute, University of Maryland, College Park, MD 20742, USA; lei@umd.edu

**Keywords:** health, built environment, urban form, socioeconomic factors, ecological model, active travel, walking, bicycling, telecommuting, teleshopping

## Abstract

As physical inactivity statistics for the U.S. population show an alarming trend, many health problems have been increasing among Americans in recent decades. Thus, identification of the factors that influence people’s physical activity levels and health outcomes has become ever more essential to promote public health. The built envSFironment is among the main factors that impact individuals’ health outcomes. However, little is known about the health impacts of built environment factors at large geographical scales such as those of the metropolitan area of residence. Further, the health impacts of travel behavior such as telecommuting and teleshopping remain unclear. This study uses an ecological model framework to probe the roles of travel behavior and built as well as social environments at different spatial levels in health. Instrumental variable binary probit models have been developed to examine the complex interlinks between measures of travel behavior, physical activity levels, built and social environment characteristics, and individuals’ health outcomes. Findings indicate that built and social environment factors at different spatial levels, including the metropolitan area, are correlated with individuals’ health outcomes. Additionally, the findings suggest that increased levels of telecommuting and teleshopping within communities may lead to unfavorable health outcomes. The findings shed light on the most promising policy interventions that can promote public health through modifications targeting people’s travel choices as well as the built and social environments within urban areas.

## 1. Introduction

**Figure 1:**
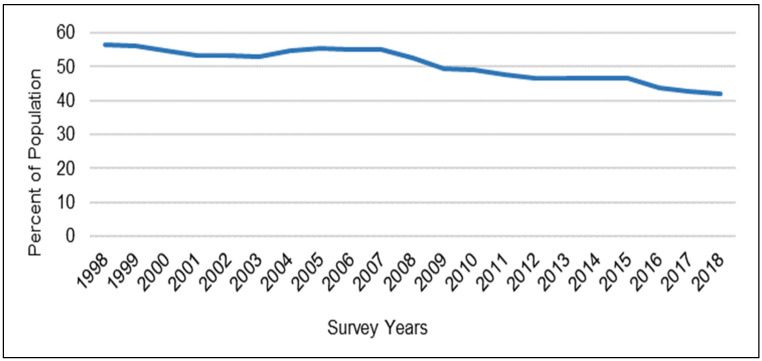
Trends in leisure-time physical inactivity among U.S. adults. Source of data: [2].

**Figure 2:**
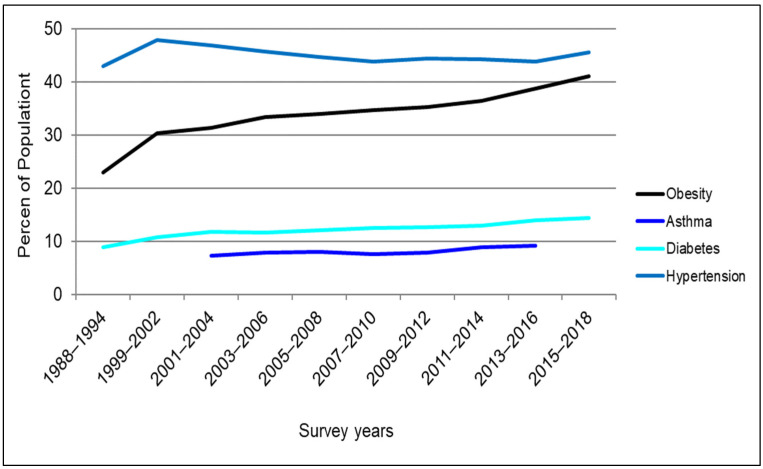
Trends in health conditions among U.S. adults. Source of data: [2,3,4,5,6].

Health is a salient life quality influencing the well-being of individuals and the vitality of communities, and for that reason, it has been a topic of great interest in many academic disciplines for many decades. Numerous endeavors have been made by researchers in the past to probe the determinants of human health. Through this research, physical activity has been identified as a key contributing factor to health [1]. Nonetheless, a snapshot of the states of physical activity levels and health for the U.S. population indicates that physical inactivity has become a trend in the U.S. (Figure 1), while obesity and other chronic health conditions have been increasing in prevalence (Figure 2).

Considering the alarming physical inactivity and public health trends in the U.S., identification of the factors that influence individuals’ physical activity levels and health outcomes has become crucial. A few of these factors include people’s travel choices and physical activity choices, as well as the built and the social environments of their residential areas.

Each of these factors, independently and in interaction with other factors, impacts individuals’ health outcomes and defines and shapes the health profiles and the overall well-being of communities.

### 1.1. The Role of Travel Behavior and Physical Activity in Health

The dominance of the mighty automobile as the main mode of transportation in U.S. cities has led to many problems including adverse environmental impacts (e.g., higher air pollution levels) and increased public health risks (e.g., higher physical inactivity levels). According to the U.S. Environmental Protection Agency (EPA) [7], transportation activities accounted for 37.5% of U.S. carbon dioxide (CO_2_) emissions from fossil fuel combustion and 28.6% of total greenhouse gas emissions in 2019. Moreover, the largest sources of transportation-related CO_2_ emissions in 2019 were passenger cars, freight trucks, and light-duty trucks [7]—i.e., the automobile. These statistics have health consequences.

Further, travel by means of the automobile restricts opportunities for physical activity, thereby leading to sedentary behaviors and lifestyles—which can, in turn, lead to adverse public health outcomes. Additional daily time spent in an automobile and longer automobile commute times have been found to be correlated with unfavorable health outcomes such as a higher body mass index (BMI) and an increased likelihood of obesity [8,9]. On the other hand, regular physical activity—including in its active travel form (i.e., walking and bicycling)—can lead to reduced risk of many health problems and diseases such as obesity, diabetes, hypertension (i.e., high blood pressure), coronary heart disease, stroke, and premature death [1]. To attain health benefits, the U.S. Department of Health and Human Services (HHS) and the Centers for Disease Control and Prevention (CDC) recommend 150 minutes of moderate physical activity (such as brisk walking or bicycling) per week for adults [1,10]. Despite the many health benefits of physical activity, physical inactivity remains a major risk factor for the health of the U.S. population. Statistics show that between 1998 and 2018, on average, nearly half of U.S. adults did not meet the recommended physical activity requirements [2], also see Figure 1. This is while the average percentage of the U.S. population that was overweight or obese increased from 56% to approximately 73% between 1988 and 2018. In the meantime, a 2018 report estimated the annual healthcare cost of lack of physical activity in the U.S. and the related adverse health outcomes to be around 117 billion dollars [1]. Thus, promoting daily physical activity is critical to improving public health and reducing healthcare costs within the U.S.

Active modes of travel such as walking and bicycling provide a great opportunity to increase physical activity levels. Walking is the oldest, simplest, and the most natural mode of transportation [11], and both walking and bicycling incorporate the added value of physical activity. The cost effectiveness and sustainability offered by these active modes of travel make them viable alternatives to driving and applicable interventions to improve individuals’ health. Health benefits of active travel are not difficult to discern, as these benefits are well-researched and well-established in the literature. The chief finding of prior research is that physical activities such as walking and bicycling are positively associated with improved health outcomes, including lower BMI and obesity rates, as well as a better general health status, e.g., [8,12,13,14,15,16,17].

The health benefits of active travel can also be extended to users of the public transit mode. This is because public transit trips involve walking at both ends for access and egress, and bicycling can also be a potentially important mode of access to public transit [18]. Increased transit use has been found to be correlated with lower obesity rates [19,20]. On the other hand, the additional walking trips associated with transit use can also expose public transit riders to polluted air and increase their risk of asthma, as found in previous research [21]. Other adverse health effects for public transit use have also been suggested, including the increased risk for diseases to spread between riders due to crowded transit vehicles [22], and lower levels of mental well-being due to undesirable interactions and the resultant negative emotions [23].

### 1.2. The Role of Built and Social Environments in Health

The main theory that effectively relates the built and social environments to health behavior, and ultimately to health, is the ecological model of behavior. As a basis for research on health behavior, the ecological model of behavior posits that factors from multiple levels of influence—namely, the individual level, the social environment level, the built environment level, and the policy levels—can affect human behavior including health behavior [24]. Within these levels, concepts that operate at multiple levels themselves are the built and social environments [24]. The ecological model puts special emphasis on the role of multiple levels of the built environment in health behavior, which can impact health outcomes. Bearing in mind the framework of the ecological model, the roles of the built and social environments in health are discussed below.

#### 1.2.1. Built Environment and Health

The relationship between the built environment and health is a rather complex one. That is due to the potential of the built environment to influence health both (*i*) directly through availability/absence of services and qualities that can improve/worsen health (e.g., access to healthy food outlets, ambient air quality), and (*ii*) indirectly through promoting health behavior (e.g., physical activity and active travel choices). Kent and Thompson [25] identified three key domains through which the built environment can influence human health: (1) physical activity; (2) social interaction; and (3) access to healthy food.

With regards to the first domain (i.e., physical activity) and within a transportation context, the dominance of the private vehicle as the main mode of travel in the U.S. can be considered as a challenge to promoting active modes of travel. This is because with the interwoven relationship between transportation and land use, high levels of automobile usage can lead to more automobile-oriented built environment designs and land development patterns, which, in turn, can result in more obstacles to using the active travel modes [26]. Where the automobile shapes the built environment and landscape in urban areas, the consequent increase in sedentary travel behavior—namely, the increased use of private vehicles and the engagement decline in active travel (i.e., physical activity)—can lead to adverse health effects for residents. A comprehensive study of the effects of the built environment on physical activity [27] provided a conceptual framework, proposing that the built environment of both the neighborhood and the region may play important roles in the amount of physical activity (e.g., walking and bicycling) performed by residents. Considering the link between physical activity and health, it can thus be hypothesized that the built environment at different spatial scales can impact individuals’ health outcomes. This argument aligns with the principles of the ecological model, which postulates that the built environment can operate at multiple levels to influence health behavior.

The second domain, social interaction, can also be facilitated (or restricted) by the built environment. For instance, a built environment that is supportive of physical activity—especially in the form of walking—can create opportunities for social interaction [28], which may have a positive health impact. Social interaction can also be influenced by the built environment in various other ways. For instance, existence of public spaces (e.g., parks, playing fields, outdoor food courts) as well as mixed-use developments can encourage individuals to spend time outside, engage in social activities, and interact with other community members—all of which, can contribute to their mental well-being and psychological health.

Further, the built environment can impact individuals’ health by facilitating or constraining the third domain—access to healthy food. An example of facilitated access to healthy food through the built environment is existence of farmers’ markets within communities, which provides access to fresh produce including farm-produced fruits, vegetables, and herbs. Studies have postulated that sprawling metropolitan areas can impact health of residents through restricting access to healthy food; see, e.g., [29].

Various aspects of the built environment have been found in prior empirical research to affect various aspects of health. For instance, higher densities (i.e., higher compactness) have been found to be associated with increased risk of asthma, hypertension, and heart attack, and to adversely affect individuals’ general health status—due to creating potentially stressful and polluted environments, as suggested in past studies [19,21,30]. Further, presence of various land uses (i.e., mixed-use development) has been found to be associated with a better general health status and a lower risk of heart attack [19]. Improved health outcomes including lower risks of obesity, diabetes, and heart disease have also been found to be associated with well-connected street network designs [31]. Smaller block sizes (i.e., proxy for higher intersection density), albeit associated with a lower risk of obesity, have been found to be also associated with increased risks of asthma and poorer general health [21]. Past empirical research also provides evidence of a correlation between destination accessibility and health outcomes. For example, higher access to fast food restaurants has been found to have a positive correlation with diabetes rates [31] and obesity rates [32].

#### 1.2.2. Social Environment and Health

The role of the social environment in individuals’ health can be represented by the socioeconomic, sociodemographic, sociocultural, and crime-related characteristics of their social settings. Considering the principles of the ecological model, one way that the social environment can impact individuals’ health status is through influencing their health behavior, such as active travel and physical activity. Social environment factors such as social norms, concerns about crime, as well as crime rates have been found to influence levels of active travel [33,34,35].

Since health is a function of health behavior such as active travel, any effect of the social environment on active travel and physical activity may have health implications. For instance, living in areas with higher violent crime rates can lead to lower active travel levels—as found in previous research, e.g., [33]—and consequently, to higher levels of obesity and/or other unfavorable health outcomes for residents. Indeed, research provides evidence for the argument that prevalence of obesity is higher in areas with more violent crime [29].

Other aspects of the social environment, such as the socioeconomic and sociodemographic attributes of the residential area including median income levels, median age, and racial composition, have also been found to be influential in health outcomes. For example, living in areas where residents have higher income levels has been found to be correlated with lower risk of developing chronic health conditions such as obesity, diabetes, and hypertension [15,31,36]. Further, higher median age has been found to be associated with higher rates of hypertension and heart attack, whereas a higher percentage of the non-white population has been found to be associated with higher rates of diabetes [31].

As with the built environment, within an ecological model framework, the social environment can exert its effects at multiple levels to influence health behavior and can thereby impact health outcomes accordingly.

### 1.3. Gaps in Research and the Study Contributions

Health promotion research is increasingly shifting toward ecological orientations, which provide more comprehensive frameworks by considering influences and interventions at multiple levels and in various contexts [25]. Research thus far asserts that human health is affected by several factors, including the travel behavior of individuals as well as the built and social environments of their surroundings. However, while many studies in the past examined the relationship between various health outcomes and built environment factors at the neighborhood and county level, little attention has been given to the health impacts of built environment characteristics at larger geographical scales such as those of the metropolitan area of residence.

To narrow that knowledge gap in research, the present study employs an ecological framework to test the hypothesis that built and social environments at different spatial levels—including the county and the metropolitan area levels—influence individuals’ health outcomes.

Further, while the health impacts of travel by modes of automobile, walking and bicycling, and public transit have been examined in past research, the role of other travel-related behavior such as telecommuting and teleshopping in health remain ambiguous, as empirical evidence in that regard is either inconsistent or insufficient.

Telecommuting may allow increased levels of physical activity by making available time that would otherwise be spent on commuting, whereas longer commutes can affect physical activity by cutting leisure times short [29]. On the other hand, physical activity levels may decrease for an individual who telecommutes due to the sedentary nature of telecommuting or because of having less time for exercise owing to the blurriness between work and personal time boundaries. This may affect the telecommuter’s health status, especially if telecommuting is performed on a regular basis over a long period of time. With respect to physical health, there have been a few hints in the literature suggesting that telecommuting can impact physical health outcomes such as various illnesses, particularly stress-related illnesses [37,38]. Nonetheless, empirical studies that investigated the role of telecommuting in physical health outcomes are scarce, and those that do exist, have produced inconsistent findings. For instance, while Henke et al. [39] found that non-telecommuters were at greater risk for obesity and physical inactivity, Tajalli and Hajbabaie [15] did not find a statistically significant association between telecommuting and obesity or other physical health indicators. Thus, previous research does not yield adequate evidence to draw conclusions about the effects of telecommuting on physical health.

Further, the effects of teleshopping behavior—a potentially influential but subtle travel-related activity—have notably been overlooked in health impact studies. Excessive online shopping can promote a sedentary lifestyle and has a potential to reduce levels of physical activity, which can affect one’s health. Studies suggest that excessive engagement in computer- and online-related activities may lead to lower physical activity levels [40,41] and contribute to weight gain [41]. Nonetheless, the role of teleshopping in health is currently an open field of research, as to the best of the authors’ knowledge, there seem to be no empirical studies on that topic.

The present study contributes to the body of knowledge by filling the gaps in empirical research regarding the health impacts of telecommuting and teleshopping. Measures of these travel behaviors as well as other travel behaviors (i.e., active, automobile, and public transit travel) at the county and metropolitan area levels have been included in this study to probe the role of travel behavior within communities in residents’ health.

## 2. Materials and Methods

### 2.1. Study Area and Data

The present study used data from several counties and metropolitan areas within the state of Florida in the U.S. (the official nickname for the U.S. state of Florida is the Sunshine State: https://www.myflorida.com/, accessed on 25 July 2022). The database for the health outcome models developed in this study consisted of the following individual datasets:

*American Community Survey* (*ACS*): The American Community Survey (ACS) is an annual survey program conducted by the U.S. Census Bureau. The survey is the leading source for detailed information about the U.S. population (e.g., socioeconomic and sociodemographic characteristics), means of commuting to work, and housing (e.g., financial and physical characteristics of housing units) at various geographical scales [42].

*Behavioral Risk Factor Surveillance System* (*BRFSS*): The Behavioral Risk Factor Surveillance System (BRFSS) is a health-related survey system that collects data from adult residents in the U.S. (and its territories) on their health behaviors, chronic health conditions, and preventive health-related practices that can influence health status. Managed by the CDC, the BRFSS is conducted monthly by state health departments as a cross-sectional telephone-based survey. The comprehensive survey design and the large number of respondents (over 400,000 adults each year) make the BRFSS the largest health survey system in the world [43]. Due to privacy concerns, however, the smallest geographical unit available for the BRFSS respondents’ addresses is the county of residence.

*Community Health Status Indicators* (*CHSI*): Available through the CDC at the time this research was conducted (the CDC is no longer updating the CHSI dataset), the Community Health Status Indicators (CHSI) dataset provided public health profiles for all 3143 counties in the U.S. The information included the health status of the population of a county as well as county-level factors that potentially affect population health outcomes (e.g., health behavior, health access, social and built environment characteristics) [44].

*County Health Rankings & Roadmaps* (*CHR&R*): The Robert Wood Johnson Foundation, in collaboration with the University of Wisconsin Population Health Institute, created the County Health Rankings and Roadmaps (CHR & R) program [45]. The CHR & R dataset provides information on county-level health profiles and rankings for each county within a specific state in the U.S. Ranking measures rank each county based on: (1) health outcomes (e.g., measures of mortality and morbidity); and (2) health factors (e.g., measures of health behavior, clinical care, social and built environments).

*National Household Travel Survey* (*NHTS*): The National Household Travel Survey (NHTS) is the most comprehensive travel survey at the national level in the U.S. The survey is periodically conducted by the Federal Highway Administration (FHWA) to collect data on the travel behavior of U.S. residents [46]. The NHTS dataset provides data on surveyed households’ geographic area, socioeconomic characteristics, and the daily trips made within a designated 24-hour period of time by households’ members. The NHTS data also provide information on other travel-related behaviors of household members such as their telecommuting and teleshopping behavior.

*Point of Interest* (*POI*) *data*: Point of Interest (POI) data contain information about the Global Positioning System (GPS) coordinates (i.e., geographic coordinates) of important locations. This study uses POI data provided by POIplaza—a publicly available GPS database that provides information for over seven million POIs (e.g., restaurants, gas stations, parking lots, banks) from 221 countries in the world [47].

*Smart Location Database* (*SLD*): A product of the U.S. Environmental Protection Agency (EPA)’s Smart Growth Program, the Smart Location Database (SLD) is a nationwide geographic dataset for the U.S. and is publicly available on the EPA web site [48]. The SLD provides information on land use and built environment characteristics such as density (for population, employment, housing), diversity of land use, neighborhood design characteristics, destination and transit accessibility, and sociodemographic characteristics at the U.S. census block group (CBG) level.

*Topologically Integrated Geographic Encoding and Referencing* (*TIGER*)*/Line Shapefiles*: An open source dataset, the U.S. Census Bureau’s TIGER/Line Shapefiles is a comprehensive dataset that provides valuable geographic information for use with Geographic Information Systems (GIS) applications. Information such as geographic boundaries, statistical geographic areas, address information, and land attributes (e.g., roads, railroads, rivers), can be obtained from this dataset [49].

*Uniform Crime Reporting* (*UCR*) *Program*: The Federal Bureau of Investigation (FBI)’s Uniform Crime Reporting (UCR) program provides reliable crime statistics for the U.S. through collecting, publishing, and archiving data on crime. The UCR program includes publicly available data that have been received from over 18,000 agencies within the U.S. [50].

*Urban Mobility Information* (*UMI*): The Texas A&M Transportation Institute (TTI) publishes the Urban Mobility Report, which uses the Urban Mobility Information (UMI) data to provide information on mobility trends within the U.S. The UMI data tables include multiyear data on the level of mobility (e.g., roadway congestion levels, travel times, annual hours of delays) for nearly 500 urban areas across the U.S. [51].

*Walk Score*: Walk Score^®^ [52] is a publicly available service that provides information on walkability of locations. A Walk Score is an objectively measured number that assesses the walkability and pedestrian friendliness of a particular address based on a destination accessibility-oriented approach. Distances to nearby walkable amenities (e.g., educational, retail, services, food, and recreational destinations) are used in an algorithm that calculates the Walk Score of a specific address. Walk Score ranges from 0 for a car-dependent (i.e., non-walkable) location to 100 for the most walkable location.

*Woods & Poole Complete Economic and Demographic Data Source* (*CEDDS*): Produced by Woods and Poole Economics, Inc., the CEDDS provides annual historical economic and demographic data for all U.S. counties, metropolitan areas, and states. These data include information such as population (total as well as by age, sex, race), employment (total as well as by industry), income, retail sales by type of business, and more [53,54].

### 2.2. Health Outcome Models: Model Framework

**Figure 3:**
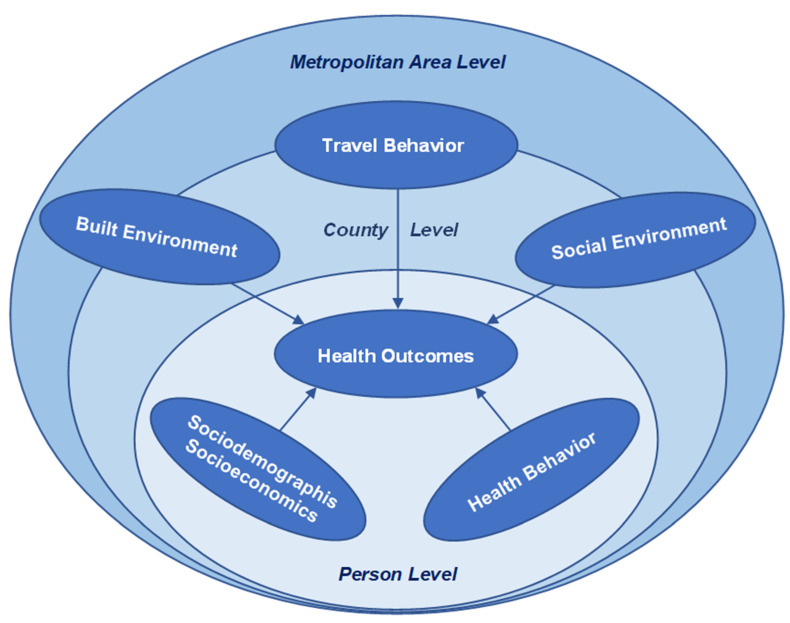
The proposed ecological model framework for the present study.

To probe the determinants of individuals’ health, this study conceptualizes the model framework shown in Figure 3, using the theory proposed by the ecological model as the model basis. To analyze individual’s health outcomes, the conceptual framework incorporates factors representing three levels of influence. These include: (1) person-level sociodemographic/socioeconomic and health behavior factors; (2) county-level built and social environments; and (3) metropolitan area-level built and social environments. Travel behavior measures at the county and the metropolitan area level have also been included in the framework.

The model dependent and independent variables are defined based on this proposed conceptual model framework (Figure 3).

### 2.3. Health Outcome Models: Dependent Variables

The dependent (i.e., endogenous) variables for the health outcome models have been selected based on the health outcomes provided in the 2009 BRFSS dataset, which represent measures of mortality and morbidity for each respondent.

Five separate models have been developed for the following five person-level health outcomes and health behavior indicators:

1. Overweight or obese;
2. Diabetes;
3. Asthma;
4. General health; and
5. Participation in 150 min (i.e., minutes) of moderate physical activity per week (as recommended by the U.S. Department of Health and Human Services (HHS) [1] and the Centers for Disease Control and Prevention (CDC) [10].

**Figure 4:**
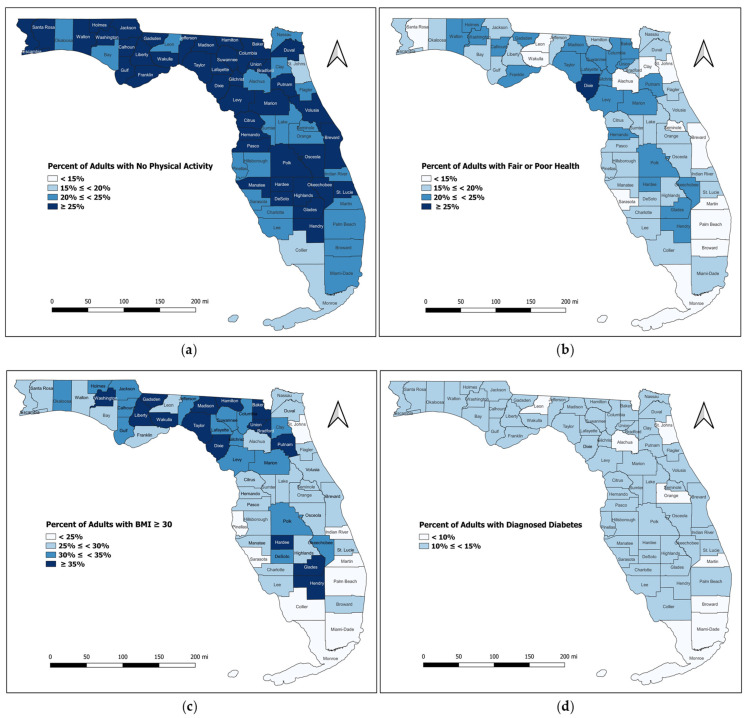
Prevalence of physical inactivity and health conditions by Florida county: (**a**) prevalence of physical inactivity; (**b**) prevalence of poor or fair health; (**c**) prevalence of obesity; (**d**) prevalence of diabetes.

Figure 4 shows the prevalence of a few of the above indicators for each Florida county based on 2009 data (obtained from the CHR & R dataset).

### 2.4. Health Outcome Models: Independent Variables

The independent (i.e., exogenous) variables for the health outcome models have been considered based on previous research (please see the Introduction section). As shown in the model conceptual framework (Figure 3), these variables represent: (*i*) health behavior and sociodemographic/socioeconomic status at the individual level (including the individual’s household characteristics); (*ii*) the built and social environments at two different spatial levels: the county and the metropolitan area of residence; and (*iii*) travel behavior at the county as well as the metropolitan area levels.

Table 1 lists the dependent and independent variables included in the health outcome models along with their descriptions, summary statistics, and data sources.

### 2.5. Health Outcome Models: Analytical Methods and Model Specification

In developing person-level health outcome models, it should be borne in mind that such analysis is susceptible to the endogeneity bias. This is due to: (*i*) the possibility of reverse causality between health outcomes and physical activity (including active travel such as walking and/or bicycling); or (*ii*) the omission of an underlying unmeasurable variable, such as internal motivation, that influences both the propensity to be physically active (e.g., engage in active travel) as well as the health status [14].

With respect to reverse causality, the assumption is that individuals’ level of physical activity impacts their health status, but the reciprocal effect can also exist: individuals’ health status may influence their levels of physical activity. This reciprocal causation effect (i.e., reverse causality) has been rationalized by researchers to exist because: (*i*) having a better health status makes individuals more likely to perform physical activity such as active travel [14]; and (*ii*) individuals with poor health may perform lower levels of physical activity [57].

With respect to omission of unmeasurable variables, the assumption is that there may exist at least one omitted underlying variable in the model that influences both an individual’s physical activity level and his/her health status. In this situation, the physical activity variable becomes an endogenous independent variable in the model—meaning that after controlling for all the other independent variables, there is a non-zero correlation between physical activity and the model’s error term. Such a correlation may lead to biased coefficient estimates in the model.

One methodology to control for endogeneity bias in estimation of models is the instrumental variable (IV) analysis. The instrumental variable method has been used in previous research as an appropriate methodology to address endogeneity bias in estimating health outcomes, e.g., [14,58], and can provide more comprehensive and less biased results.

Thus, to account for any potential endogeneity bias in the person-level health outcome models developed in the present study, the instrumental variable analysis approach has been employed. The instrumental variables in the health outcome models have been used for the endogenous physical activity independent variable (i.e., the *Physical activity minutes* variable) in models with a health-related dependent variable (i.e., the *Overweight or obese*, *Diabetes*, *Asthma*, *Good general health* variables). No endogeneity was assumed in the model with the physical activity-related dependent variable (i.e., the *CDC physical activity* variable) due to no assumption of reverse causality in that model.

The health outcome variables in this study are in a binary form, indicating the diagnosis (or lack thereof) of a specific health outcome for an individual. This requires the employment of binary choice models. As in many past studies, e.g., [20,21], binary probit modeling has been selected in the present study as the statistical technique to model the health outcomes. The person-level instrumental variable binary probit health models have been formulated as:

(1) $$y_{1i}^{*}=c_{1i}+\beta y_{2i}{}_{\;}+\gamma_{1i}^{\prime }{\mathrm{ED}}_{P\;}+\gamma_{2i}^{\prime }{\mathrm{BE}}_{C}+\gamma_{3i}^{\prime }{\mathrm{BE}}_{\mathrm{MA}}+\gamma_{4i}^{\prime }{\mathrm{SE}}_{C}+\gamma_{5i}^{\prime }{\mathrm{SE}}_{\mathrm{MA}}+\gamma_{6i}^{\prime }{\mathrm{TB}}_{C}+\gamma_{7i}^{\prime }{\mathrm{TB}}_{\mathrm{MA}}+\mu_{i}$$

(2) $$\;y_{2i}=c_{2i}+\Pi_{1}{\mathrm{ED}}_{P}+\Pi_{2}{\mathrm{BE}}_{C}+\Pi_{3}{\mathrm{BE}}_{\mathrm{MA}}+\Pi_{4}{\mathrm{SE}}_{C}+\Pi_{5}{\mathrm{SE}}_{\mathrm{MA}}+\Pi_{6}{\mathrm{TB}}_{C}+\Pi_{7}{\mathrm{TB}}_{\mathrm{MA}}+\Pi_{8}Z_{P}+\vartheta_{i}$$

where

$y_{1i}^{*}=$ value of the unobserved endogenous person-level health outcome variable (*i*);

$y_{2i}=$ value of the observed endogenous person-level physical activity variable (for outcome (*i*);

$c_{1i},\;c_{2i}$ = model intercepts;

β= model structural parameter for the instrumented endogenous variable (i.e., the *Physical activity minutes* variable);

$\gamma_{1i\;}^{\prime }-\gamma_{7i\;}^{\prime }=$ column vectors of model structural parameters;

$\Pi_{1}-\Pi_{8}=$ matrices of reduced-form parameters;

${\mathrm{ED}}_{P\;}=$ column vector of exogenous person-level (and person’s household) attributes;

${\mathrm{BE}}_{C\;}$ and ${\mathrm{BE}}_{\mathrm{MA}\;}=$ column vectors of exogenous county-level and metropolitan area-level built environment attributes, respectively;

${\mathrm{SE}}_{C\;}$ and ${\mathrm{SE}}_{\mathrm{MA}\;}=$ column vectors of exogenous county-level and metropolitan area-level social environment attributes, respectively;

${\mathrm{TB}}_{C\;}$ and ${\mathrm{TB}}_{\mathrm{MA}\;}=$ column vectors of exogenous county-level and metropolitan area-level travel behavior attributes, respectively;

$Z_{P\;}=$ column vector of additional person-level instrumental variables;

$\mu_{i}\;\mathrm{and}\;\vartheta_{i}=$ model error terms; and

*i*= (overweight or obese, diagnosed with diabetes, diagnosed with asthma, good general health).

It should be noted that the equation for $y_{2i\;}$(Equation (2) is written in the reduced form. Also, by assumptions of the model, ($\mu_{i},\vartheta_{i}$) ∼ N (0, Σ), where $\sigma_{11}$ is normalized to one to identify the model. The model is derived under the assumption that ($\mu_{i},\vartheta_{i}$) is iid multivariate normal for all observations (in the model for health outcome *i*). In addition, while $y_{1i}^{*}$ is not observable, the observed value of the endogenous health outcome variable, $y_{1i\;}$, changes as follows:

$$y_{1i\;}=\{\;\begin{array}{c} 0\;\;\;\;\;if\;\;\;\;\;\;y_{1i}^{*}<0\;\\ 1\;\;\;\;\;if\;\;\;\;\;\;y_{1i}^{*}\;\geq 0\; \end{array}$$

Thus, the instrumental variable analysis formulated above consists of two stages: in the first stage, the endogenous independent variable (i.e., the *Physical activity minutes* variable) is modeled as a function of control variables plus the instrumental variables, Z_P_ (which are assumed to be strongly correlated with the *Physical activity minutes* variable but have zero correlation with the error term in the health outcome model). In the second stage, the observed value of the *Physical activity minutes* variable is replaced with its predicted value obtained from the first stage of analysis, and the health outcome binary probit model is subsequently estimated based on the predicted values of the *Physical activity minutes* variable plus the control variables. The instrumental variable analysis isolates the variation in the instrumented variable (i.e., the *Physical activity minutes* variable) that is not due to reverse causality or omitted variables, thereby achieving a more accurate estimate for its coefficient in the model [14].

According to Wooldridge [59], the instrumental variables contained in column vector Z must satisfy two conditions: (1) exogeneity; and (2) correlation. Exogeneity implies that the instrumental variable must be uncorrelated with the error term in the health outcome models ($\mu_{i}$). Correlation implies that the instrumental variable must be partially correlated with the endogenous independent variable (i.e., the *Physical activity minutes* variable, $y_{2i}$).

Considering these conditions, three person-level variables have been selected as instrumental variables in this analysis:

- *Employment status* (1: individual is employed, 0: otherwise);
- *College education* (1: individual has a college degree, 0: otherwise); and
- *Children* (number of children under 18 years of age in individual’s household).

These variables have been chosen as instrumental variables because: (*a*) they can be correlated with an individual’s propensity for and level of physical activity, which satisfies the correlation condition; and (*b*) they should not theoretically be correlated with the error term in the health outcome models ($\mu_{i}$) after controlling for the other factors, which satisfies the exogeneity condition.

With regards to item (*b*) above, findings of past research are inconsistent in terms of existence of a correlation between individuals’ health outcomes and their educational attainment, employment status, or living-with-children status. For instance, while some studies suggested that individuals’ level of education is significantly correlated with BMI [60] and being obese [8,21], others found no significant correlation between individuals’ education and being obese or having asthma [19]. Additionally, no significant correlation was found between having children and being obese or having asthma [19,21]. Further, although both of the latter studies found a correlation between having children and individuals’ general health condition, their findings should be treated with caution due to inconsistency in the direction of effects. The direction of the correlation between having children and individuals’ general health was found to be negative in the first study [19], whereas it was found to be positive in the second one [21]—making the end result inconclusive. Overall, the inconsistencies in past findings suggest that educational status, employment status, and number of children may not have any correlations with the error term in the specific health outcomes of interest in this analysis (i.e., obesity, diabetes, asthma, and general health) and can thus be considered suitable instrumental variables to model these health outcomes.

## 3. Results and Discussion

Table 2 summarizes the estimation results of the instrumental variable (IV) binary probit models developed in the present study for the person-level health outcomes. Average marginal effects have been computed for each independent variable to facilitate interpretation of the estimated coefficients. Table 3 provides the average marginal effects, estimating the probability of the individual being overweight or obese, having been diagnosed with diabetes, having been diagnosed with asthma, having a good or excellent health status (the individual reporting a “good or better” health status on their 2009 BRFSS survey responses), and meeting the CDC-recommended physical activity levels.

The results presented in Table 2 and Table 3 show that individuals’ health status indicators are linked to:

- Individual- and household-level characteristics;
- Built environment attributes of the residential area at different spatial levels (i.e., county and metropolitan area);
- Social environment attributes of the residential area at different spatial levels (i.e., county and metropolitan area); and
- Travel behavior attributes (i.e., active travel, private vehicle travel, public transit travel, telecommuting, and teleshopping) of the residential area at different spatial levels (i.e., county and metropolitan area).
- Section 3.1, Section 3.2, Section 3.3, Section 3.4 and Section 3.5 elaborate on the results of the health outcome models and also discuss them in the context of previous findings.

### 3.1. Person-Level Variables (Individual and Household Attributes): Findings

The results of the models emphasize the influential role of attributes related to individuals and their households in person-level health outcomes. These characteristics represent the sociodemographic and socioeconomic status of the Florida 2009 BRFSS respondents as well as their health behavior in the models.

Not surprisingly, older age is associated with adverse health outcomes, including a higher likelihood of having been diagnosed with asthma, a higher likelihood of having been diagnosed with diabetes, a lower likelihood of having good or excellent general health, and a lower likelihood of meeting the CDC’s recommendations on physical activity levels (i.e., participation in 150 min of moderate physical activity per week). The coefficient of the *Age* variable also has a positive association with being overweight or obese (Table 2), although the average marginal effect is not statistically significant for this variable (Table 3). These findings are consistent with those of the past studies suggesting that older age is associated with poorer health outcomes, e.g., [8,9,15,19,29,60,62], and with a lower likelihood of participation in physical activity as well as lower levels of physical activity, e.g., [29,63,64,65].

Additionally, race proves to be an influential factor in individuals’ health outcomes. The results show that being of the white race is associated with an increased likelihood of participating in at least 150 min of physical activity per week, an increased likelihood of having a good or excellent general health status, and a lower likelihood of having been diagnosed with diabetes or being overweight or obese. These findings are in line with those of previous studies suggesting that the likelihood of engaging in physical activity and meeting the recommended physical activity levels are greater for white individuals than other races, e.g., [12,63,65], and that being of the white race is generally associated with having better health outcomes, especially with respect to obesity and diabetes, e.g., [12,29,57].

Further, the individual’s gender plays a key role in his/her health outcomes and physical activity levels. The results presented in Table 2 and Table 3 indicate that males are more likely to meet the CDC’s recommendations on physical activity levels. Results show that males are also more likely to be overweight or obese, but they have a lower likelihood of being diagnosed with asthma (see Table 3: the average marginal effects are significant in the *Overweight or obese* and the *Asthma diagnosis* models), and perhaps have a lower likelihood of being diagnosed with diabetes (see Table 3: the average marginal effect is not statistically significant in the *Diabetes diagnosis* model). Lending support to these findings are many previous studies suggesting that males are more likely to be physically active and typically have higher physical activity levels, e.g., [12,29,63,64,65]. Additionally, several studies found that BMI was higher for males than for females, e.g., [12,29,60], and that diabetes and other health problems were more prevalent in males than females [29]—all of which are consistent with findings of the present study.

Results also show that being employed is associated with increased likelihood of having a good or excellent general health status, and higher education is related to a higher likelihood of meeting the CDC’s recommendations on physical activity levels as well as a higher likelihood of having good or excellent general health. These results are consistent with previous findings suggesting that higher levels of educational attainment are associated with a higher likelihood of engaging in physical activity as well as with higher levels of measured physical activity, e.g., [12,63,65]. The results are also consistent with studies that found higher levels of educational attainment at the person level were associated with physical healthiness [15], and with those that reported county-level higher educational attainment measures were associated with lower prevalence of diabetes [66].

Further, increased household income levels are linked with favorable health outcomes. Higher household incomes show positive associations with a lower likelihood of being diagnosed with asthma or diabetes (average marginal effects are statistically significant for all income groups in the *Asthma diagnosis* model and for the highest income group in the *Diabetes diagnosis* model). Higher incomes are also positively associated with an increased likelihood of meeting the CDC-recommended physical activity levels and having good or excellent general health. These results corroborate past findings that indicated higher incomes were associated with higher levels of physical activity, e.g., [29,63], better health outcomes, and a better general health status, e.g., [8,15,19,21,29]. The results imply that lower-income households may bear a greater burden of health disparities.

Moreover, the greater the number of children in an individual’s household, the higher the likelihood of that individual having a good or excellent general health status. This result is consistent with findings of Samimi and Mohammadian [21], who reported that individuals’ general health was positively correlated with having children. However, this finding stands in contrast with findings of Langerudi et al. [19], who found a negative correlation between general health and having children. The inconsistencies in these findings suggest that further research may be needed to elucidate the role of having children or living with children in individuals’ general health.

Among other key factors that impact individuals’ health outcomes are factors representing their health behavior. In this study, individuals’ health behavior has been controlled for by including variables representing their fruit and vegetable consumption, alcoholic beverage consumption, smoking status, and physical activity levels. These factors are postulated to be influential in personal health and well-being, and several prior studies have considered some of them in their analysis, e.g., [8,15,29,67].

Based on the results presented in Table 2 and Table 3, more servings per day of fruits and vegetables are linked with having more favorable health outcomes including a lower likelihood of being overweight or obese and a higher likelihood of having a good or excellent general health status. Moreover, a higher likelihood of meeting the CDC’s recommendations on physical activity levels is associated with higher consumption levels of fruits and vegetables. Lower probabilities of being diagnosed with asthma and diabetes may also be associated with higher consumption levels of fruits and vegetables (although the average marginal effects are not statistically significant in the *Asthma diagnosis* and the *Diabetes diagnosis* models). These findings corroborate those of past research, which reported that lower BMI was associated with higher daily levels (i.e., three or more servings) of consumption of fruits and vegetables [8,29] and that having a healthy diet was associated with better health outcomes [15].

The smoking status of the individual also proves a crucial factor in individual’s health outcomes. Results indicate that compared to nonsmokers, individuals who smoke cigarettes (i.e., everyday smokers and someday smokers) have a lower likelihood of being overweight or obese and may have a lower likelihood of having diabetes. Albeit somewhat counter-intuitive, these results corroborate past research that found BMI and obesity levels were lower for smokers compared to nonsmokers [8,29,60]. As expected, however, current smokers suffer from adverse health effects including a higher likelihood of being diagnosed with asthma and a lower likelihood of having good or excellent general health. Current smokers also are less likely to meet the CDC-recommended physical activity levels, which is consistent with findings of prior research suggesting that participation in physical activity and measured physical activity levels were lower among smokers compared to nonsmokers [8]. Former smokers, on the other hand, have a higher likelihood of being overweight or obese and a higher likelihood of being diagnosed with diabetes compared to individuals who have never smoked (i.e., the nonsmoker category, which is the base category in the models). Compared to nonsmokers, former smokers are also more likely to have been diagnosed with asthma and are less likely to have a good or excellent general health status. These results suggest that smoking, even if only in the past, has adverse health effects—an anticipated finding.

The drinking status of individuals, which represents the level of consumption of alcoholic beverages by them, shows a positive association with increased likelihood of having been diagnosed with diabetes. This implies that as the number of alcoholic beverages consumed per month by the individual increases, so may his/her risk of developing diabetes. These findings are in line with the literature suggesting that high levels of liquor intake are associated with increased risk of diabetes [68,69,70].

As regards physical activity, the results show that higher levels of moderate physical activity (i.e., more minutes per week) are linked with better health outcomes, including a lower likelihood of being overweight or obese, a lower likelihood of having been diagnosed with asthma and/or diabetes, as well as an increased likelihood of having a good or excellent general health status. These findings corroborate past findings, which suggested that: (*i*) participation in physical activity was associated with better health outcomes including better general health, a lower likelihood of obesity, and a lower likelihood of asthma [19]; (*ii*) higher physical activity levels were associated with lower probabilities of being obese and having diabetes [15]; and (*iii*) higher levels of physical activity in the form of active travel were correlated with lower BMI, a lower likelihood of being overweight/obese and having other adverse health outcomes as well as an increased likelihood of having good general health [14].

### 3.2. Built Environment Variables: Findings

In this study, the effects of the built environment on person-level health outcomes have been controlled for at two spatial levels: the county level and the metropolitan area level. The county- and the metropolitan area-level built environment variables represent the five *D*s of the built environment—namely, density, diversity, design, distance to transit, and destination accessibility—in the health outcome models. Basically, these variables characterize each Florida county and/or metropolitan area based on levels of compactness (i.e., activity density), land use diversity (i.e., entropy), design of the street network in terms of connectivity and walkability, access to local transit (i.e., distance to transit), employment destination accessibility, and level of mobility (i.e., roadway congestion levels). Moreover, each county has further been characterized based on other destination accessibility factors (i.e., access to clinical healthcare, access to recreational facilities, access to healthy and unhealthy food outlets), as well as ambient air pollution. The model coefficient estimates and average marginal effects show that many of the *D*s of the built environment play important roles in the health status of Florida residents.

#### 3.2.1. Density

The results indicate that despite being positively associated with higher probabilities of the individual meeting the CDC’s recommendations of participating in at least 150 min of moderate physical activity per week, the county-level *Mean activity density* variable (which represents compactness in terms of employment and housing density) shows positive associations with the probability of the individual being overweight or obese and the probability of having been diagnosed with diabetes. In addition, this variable is negatively associated with the probability of having a good or excellent general health status.

Moreover, the metropolitan area-level *Mean activity density* variable shows positive associations with the probability of the individual being overweight or obese, and the probability of having been diagnosed with diabetes. The average marginal effect for this variable is only statistically significant in the *Overweight or obese* model. Nonetheless, the directions of the effects are consistent with those for the county-level *Mean activity density* variable and corroborate those findings.

The results imply that despite their potential pedestrian- and bicyclist-friendly designs and providing a better opportunity for physical activity, higher densities (i.e., higher compactness)—as suggested by many past studies—may adversely affect individuals’ health due to creating potentially stressful environments [19,21,30]. Although, a few previous studies found that residents of more compact counties have lower BMIs [8,29,60] as well as lower probabilities of obesity and diabetes [29], it should be borne in mind that those studies operationalized compactness based on a sprawl index, which combined factors representing different dimensions of the built environment (i.e., density, land use diversity, centering of jobs and population, and street network design). Thus, further research may be needed based on consistent definitions to clarify the role of compactness (i.e., density) in obesity and diabetes.

Additionally, the effect of both the county- and metropolitan area-level activity density variables on asthma diagnosis shows a negative direction, indicating that a lower probability of asthma is associated with an increased activity density within the area of residence. This result is counter-intuitive as based on past research [19], one would expect dense urban areas to be the more polluted ones—residing in which may lead to more respiratory health problems such as asthma. Therefore, further research is also warranted to better understand the role of density levels within an area in asthma diagnosis of residents.

#### 3.2.2. Diversity of Land Use

Table 2 and Table 3 indicate that the county-level *Mean entropy* variable (which captures the extent of land use diversity within a county) has a positive association with the probability of meeting the CDC’s physical activity recommendations and a negative association with the probability of being overweight or obese. The coefficient and the average marginal effect for this variable show a statistically insignificant effect in the probability of having a good or excellent general health status.

Results further indicate that as its counterpart at the county level, the metropolitan area-level *Mean entropy* variable (which represents the extent of land use diversity within a metropolitan area) shows a positive association with the probability of meeting the CDC’s physical activity recommendations. This variable is also positively associated with the probability of reporting good or excellent general health. This means that a higher extent of mixed-use development within metropolitan areas is associated with an increased likelihood of higher levels of physical activity and better general health status for residents. These results are somewhat in line with past findings such as those of Langerudi et al. [19], who found that presence of various land uses within a neighborhood was associated with better general health.

On the other hand, both the county- and metropolitan area-level *Mean entropy* variables exhibit a positive association with the probability of having been diagnosed with asthma and/or diabetes. With respect to asthma, the results may be capturing the effects of availability of additional destinations. As mixed land use within a county or metropolitan area increases, additional destinations become available at remoter locations, which may encourage additional vehicular trips. The increased number of vehicular trips may lead to higher pollution levels within the county or metropolitan area, thereby increasing the risk of asthma for residents.

#### 3.2.3. Design of Street Network

Higher levels of county-level intersection density (proxy for street network connectivity) are associated with lower probabilities of meeting the CDC’s physical activity recommendations. It should be borne in mind that the *Mean intersection density* variable in this study represents intersection density in terms of automobile-oriented intersections (see Table 1); therefore, this finding is expected. An increased number of automobile-oriented intersections may mean fewer pedestrian and bicyclist facilities, which can lead to lower levels of active travel and other forms of physical activity. Nonetheless, this variable does not show a statistically significant effect on being overweight or obese. This result is consistent with that of Samimi and Mohammadian [21] but stands in contrast with the findings of Samimi et al. [20], who reported a negative association between obesity and county-level intersection density. The inconsistent findings warrant further research into the role of intersection density at the county level in individuals’ health outcomes, particularly weight-related outcomes such as obesity.

Model estimates (Table 2) and average marginal effects (Table 3) also indicate that increased intersection density throughout the county as well as the metropolitan area is associated with a higher probability of having a better general health status. This is while previous research did not find a statistically significant association between intersection density and the status of the population’s general health [20,21]. Therefore, the results obtained here should be treated with caution, and further research may be needed to clarify the role of intersection density in individuals’ general health status. On another note, the results indicate that intersection density at the metropolitan area level may be more influential in general health than at the county-level. In other words, compared to those of the county-level *Mean intersection density* variable, the coefficient estimates and average marginal effect for the metropolitan area-level *Mean intersection density* variable are larger in magnitude in the *Good general health* model. This means that better general health status is associated with living in compact metropolitan areas (in terms of intersection density), which is partly consistent with findings of Marshall et al. [31], who suggested that city-level intersection density was more important in determining health outcomes than the same variable at the neighborhood level. This, as the referenced study also suggested, can mean that better general health outcomes may be associated with residing in a more compact, better connected city than a compact neighborhood surrounded by a sparse city [31].

Further, the average marginal effects computed for both the county- and metropolitan area-level *Mean intersection density* variable (Table 3) indicate that increased intersection densities throughout the county and metropolitan area are associated with higher probabilities of being diagnosed with asthma. Since the *Mean intersection density* variable in this study represents intersection density in terms of automobile-oriented intersections (see Table 1), these results are reasonable. More automobile-oriented intersections within an area can mean higher rates of automobile use and higher levels of air pollution due to vehicle emissions—an externality that may lead to lung diseases [71] such as asthma. In addition, a previous study found that smaller average block sizes (proxy for higher intersection density) were related to higher asthma rates [21], which is consistent with findings of the present study.

#### 3.2.4. Distance to Transit

The model estimates (Table 2) and the average marginal effects (Table 3) of both the county-level and the metropolitan area-level *Mean local transit accessibility* variables suggest that increased distances to the nearest local transit stop are associated with lower probabilities of having been diagnosed with asthma. On the other hand, increased distances to local transit stops are also correlated with adverse health effects such as a higher likelihood of being overweight or obese, a higher likelihood of having been diagnosed with diabetes, and a lower likelihood of having a good or excellent general health status. Residential areas farther away from transit stops are most likely the sprawled suburban areas, which provide cleaner air to breath and thereby can lower the probability of an asthma infection. However, these suburban areas typically do not provide many opportunities for walking and bicycling. This can lead to lower levels of active travel, which, in turn, may lead to higher levels of obesity and other health problems for residents. As the literature suggests that sprawl is related to higher obesity rates, e.g., [29,72,73], these results imply that living in sprawled suburban areas with lower levels of access to transit may lead to obesity and other adverse health effects.

With regards to physical activity, it can be seen from Table 2 and Table 3 that an increased distance to transit stops is associated with a higher probability of participating in at least 150 min of moderate physical activity per week. This result may seem counter-intuitive at first, since one would expect to see lower levels of physical activity—such as active travel—performed by residents of suburban areas, which are also typically areas located farther away from transit stops. However, it should be borne in mind that the BRFSS defines moderate physical activity as activities including “brisk walking, bicycling, vacuuming, gardening, or anything else that causes some increase in breathing or heart rate” [55]. Therefore, the results might be an indication of residents of suburban areas engaging in other physical activities (e.g., gardening), and not necessarily active travel.

#### 3.2.5. Destination Accessibility

##### Regional Accessibility to Employment

The results for the *Mean temporal automobile accessibility* variables (Table 2 and Table 3) at both the county and the metropolitan area levels suggest that higher regional accessibility to jobs by means of automobile is associated with an increased probability of residents being overweight or obese, a lower likelihood of participating in at least 150 min of moderate physical activity per week, and a lower probability of reporting good or excellent general health. These results can be capturing the effect of long commutes. Longer commutes by means of automobile can mean additional commute-related stress and/or lower levels of physical activity—both of which can lead to obesity and other adverse health effects. Previous studies suggest that long commutes limit physical activity levels by cutting into leisure time [29] and can have adverse health effects on human health over time due to a number of reasons including fatigue and elevated stress levels related to operating and navigating the vehicle during rush-hour traffic, driving on congested roadways, and having to deal with aggressive driving behavior such as road rage [74,75,76,77,78].

The results for the *Mean temporal transit accessibility* variables at both the county and the metropolitan area levels suggest that higher regional accessibility to jobs by means of transit is associated with a lower probability of being overweight or obese. These results are in line with findings of a previous study, which suggested that increased transit use can lead to lower obesity rates [19,20]. Further, Table 2 and Table 3 show that at the metropolitan area level, this variable is associated with increased probabilities of participating in at least 150 min of moderate physical activity per week and reporting good or excellent general health. The physical activity associated with transit use can lead to lower levels of obesity. The literature suggests that transit accessibility can promote active travel [79,80], as most transit users arrive at and depart from transit stops via walking [81].

Table 2 also indicates that increased levels of regional accessibility to jobs by both automobile and transit may be associated with an increased likelihood of asthma diagnosis. These results are expected, as increased levels of accessibility to jobs by automobile and transit can mean increased levels of use of these modes for commuting. Increased use of automobiles means higher levels of vehicle emissions, which can affect respiratory health and lead to asthma. Widespread car usage has been suggested to lead to increasing rates of asthma in past research [74]. With respect to transit, the results of the present study confirm the findings of a previous study that found a significant, positive association between increased use of transit and being diagnosed with asthma [21]. Considering that walking is the main mode of travel for accessing/egressing transit stops [81], one reason for a positive association between higher transit accessibility and higher rates of asthma can be the additional exposure to harmful particles in the air due to the increased time spent outdoors when walking to and from transit stops or while waiting for the vehicle to arrive. Increased walkability of an area has been previously linked to higher levels of traffic-related pollution [82]. Thus, additional walking due to additional transit use may lead to inhaling increased amounts of harmful vehicle emissions, which can lead to asthma. Past studies suggest that transit riders are more exposed to polluted air due to the additional walking associated with transit use as well as the mechanical friction processes involved in transit vehicle operations, and, therefore, they are at higher risks of having asthma [21,23]. With regards to average marginal effects (Table 3), only the county-level *Mean temporal transit accessibility* variable reaches a statistical significance threshold in the *Asthma diagnosis* model; hence, caution should be exercised in drawing inferences from these results.

In addition, the model coefficient (Table 2) of the metropolitan area-level *Mean temporal automobile accessibility* variable and the average marginal effect (Table 3) of the county-level *Mean temporal automobile accessibility* variable in the *Diabetes diagnosis* model are statistically significant, suggesting that increased automobile accessibility to jobs is associated with a higher likelihood of being diagnosed with diabetes. The coefficient estimates (Table 2) of both the county- and the metropolitan area-level *Mean temporal transit accessibility* variables in the *Diabetes diagnosis* model are also statistically significant, suggesting that increased transit accessibility to jobs may be associated with a lower likelihood of being diagnosed with diabetes. It should be noted, however, that the average marginal effects (Table 3) of these variables do not reach a statistical significance threshold; therefore, additional research may be needed to clarify the role of regional accessibility to jobs by means of transit in being diagnosed with diabetes.

##### Local Accessibility to Amenities

The model estimation results for the *Mean Walk Score* variable, which is a measure of accessibility to local destinations/amenities as well as walkability [52], provide evidence that living in a county with a higher Walk Score is associated with a lower likelihood of having undesirable health outcomes (e.g., being overweight or obese) and a higher likelihood of having a good or excellent general health status. These results corroborate findings of past research, e.g., [13], and highlight the importance of higher levels of accessibility to local destinations (e.g., shops, banks, schools) as well as walkability within the residential area in the health status of individuals.

##### Access to Healthy/Unhealthy Food Outlets

Table 2 and Table 3 provide evidence for the role of the food environment in human health. The results show that higher densities of fast food restaurants within the county are associated with a higher likelihood of being obese or overweight, having been diagnosed with asthma and/or diabetes, and with a lower likelihood of meeting the CDC’s recommendations on physical activity and having a good or excellent general health status. With respect to obesity, these results corroborate the findings of past research [32]. Moreover, the results provide evidence for arguments by: (*i*) Joshu et al. [57], who suggested that physical environments that promote unhealthy food choices can also promote obesity; (*ii*) Plantinga and Bernell [60], who suggested that prevalence of fast food restaurants is one of the factors that may explain the rise in obesity rates in the U.S.; and (*iii*) Croucher et al. [83], who, after conducting a review of literature, suggested that empirical evidence supports a link between the density of fast food outlets and obesity. Considering diabetes, at least one previous study found that more fast food restaurants within the city were associated with higher diabetes rates [31]—a finding in line with findings of the present study.

In contrast and as expected, access to healthy food outlets seems to have a favorable influence on health outcomes. Results show that increased levels of access to healthy food outlets within the county are associated with a lower likelihood of being obese or overweight and having been diagnosed with asthma and/or diabetes and a higher likelihood of meeting the CDC’s physical activity recommendations and having a good or excellent general health status. It should be noted that the average marginal effect of the *Access to healthy food outlets* variable in the *Overweight or obese* model does not reach a statistically significant threshold, which means that further examination of the role of access to healthy food in weight-related health outcomes may be needed. Nevertheless, these results imply that access to healthy food plays an important role in individuals’ health and confirm arguments by past studies suggesting that access to healthy food can influence health outcomes [8,71]. The findings provide further evidence that access to healthy food—as suggested by Kent and Thompson [25]—is one of the main domains through which the built environment influences human health. Although access to healthy food may be a challenge in sprawling environments [29], the results of the present study imply that lack of or limited access to healthy food can lead to poor health outcomes. Therefore, access to healthy food is a key element in retaining good health.

##### Access to Parks

Living in the vicinity of parks seems to be associated with a lower likelihood of being overweight or obese (bear in mind, however, that the corresponding average marginal effect is not statistically significant, as shown in Table 3), and a lower likelihood of having been diagnosed with diabetes, as well as a higher likelihood of meeting the CDC’s physical activity recommendations. These results are reasonable, as living near parks can encourage people to get out of their houses, exercise, and enjoy the outdoors—which can lead to a better state of health. The literature suggests that lower BMI is associated with more of the county land devoted to parks [29] and that access to green spaces can affect health [71]. The results of the present study confirm those hypotheses. However, based on the results, access to parks is also associated with a higher likelihood of being diagnosed with asthma. This is a reasonable finding, as spending time near plants and vegetation increases exposure to environmental triggers and allergens such as pollen. Exposure to pollen may lead to respiratory allergic illness and/or exacerbation of asthma [84].

##### Access to Healthcare Providers

Better access to clinical care within the county seems to contribute to a better health status for residents. The results indicate that an increased number of primary care physicians per 100,000 county population is associated with a lower likelihood of the residents being overweight or obese. In addition, higher levels of access to clinical care within the county are associated with residents reporting a good or excellent general health status. These results further support the hypothesis that increased access to healthcare within an area contributes to the betterment of the health status of residents.

#### 3.2.6. Other Built Environment Attributes

##### Ambient Air Pollution Levels

Exposure to air pollution—particularly transportation- and traffic-related air pollution—can adversely affect health [82,85,86]. The results of the present study provide support for this argument. As seen in Table 2 and Table 3, the *Ambient air pollution* variable (which represents the annual number of ambient air pollution days within the county of residence due to ozone and fine particulate matter) has unfavorable, statistically significant associations with some of the health outcomes. Most notably, and as expected, an increased number of air pollution days is positively associated with the probability of having been diagnosed with asthma. This result is consistent with the literature suggesting that air pollution is related to asthma and that outdoor air pollution exacerbates asthma in individuals who already have the condition [84]. Other results suggest that increased exposure to air pollution is associated with a lower probability of having good or excellent general health. Together, these findings are supported by statements in a report published by the World Health Organization [85] suggesting that air pollutants such as ozone and fine particulate matter are associated with adverse health effects.

##### Mobility Levels

The *Mean roadway congestion index* variable, which in this study represents the levels of mobility within the metropolitan area of residence, is positively associated with the probability of being obese or overweight (Table 3). This result is consistent with findings of Joshu et al. [57], who reported that heavy traffic was moderately associated with obesity among residents of large metropolitan and micropolitan areas. Additionally, this variable is positively associated with the probability of having asthma (Table 3), and it is negatively associated with having good or excellent general health status as well as meeting the CDC’s recommendations on physical activity (Table 2 and Table 3).

These results imply that higher congestion levels (i.e., lower mobility levels) within metropolitan areas of residence may adversely impact the health status of residents. The two main culprits are likely to be: (1) higher levels of stress in residents of such congested urban areas; and (2) less time to engage in physical activity for car commuters who drive on congested roadways. This argument is supported by past studies suggesting that driving on congested roads is a contributor to stress, e.g., [23,74,87,88,89]. In addition, higher congestion levels may be an indication of longer automobile commute times, which, based on the literature, may lead to increased levels of stress in commuters [76] as well as lower levels of physical activity [29] and thereby can have adverse health effects [74,89], including obesity. Furthermore, past research provides evidence that: (*i*) additional daily time spent in an automobile is associated with an increased likelihood of obesity [8]; and (*ii*) a longer duration of car commuting is associated with a higher BMI [9]. Concerning asthma, it should be noted that increased congestion levels mean additional exposure to harmful air pollutants due to vehicle emissions, which can lead to respiratory health problems such as asthma. Past research provides support for this argument by suggesting that: (*i*) increased car usage may lead to increased rates of asthma [74]; and (*ii*) residential proximity to heavy traffic increases the risk of asthma occurrence and exacerbation [90].

### 3.3. Social Environment Variables: Findings

Like those of the built environment, the effects of the social environment on person-level health outcomes have been accounted for at two spatial levels: the county level and the metropolitan area level. These variables characterize each Florida county and/or metropolitan area based on sociodemographic, socioeconomic, and crime-related factors. The model coefficient estimates and average marginal effects show that several social environment factors play key roles in health outcomes of Florida residents.

#### 3.3.1. Sociodemographic Attributes

The *Median age* variable, which represents the median age of the county population, shows a positive correlation with the likelihood of having been diagnosed with asthma and having been diagnosed with diabetes (although the average marginal effect is not statistically significant in the case of diabetes). In addition, this variable exhibits a negative correlation with the likelihood of having a good or excellent general health status and meeting the CDC’s recommendations on physical activity levels. These results are consistent with those obtained for the *Age* variable (which represents the age of the respondents) and further indicate that older age is associated with lower levels of physical activity, as well as with adverse health outcomes—a finding that corroborates those of past research, e.g., [9,19,29,64].

Results also suggest that the racial composition of the county plays a role in the health outcomes for residents, as having a higher percentage of white residents within the county is associated with an increased likelihood of participating in at least 150 min of physical activity per week and an increased likelihood of having a good or excellent general health status. These findings are consistent with those of previous studies, which suggested that being white was associated with a higher likelihood of engaging in physical activity and meeting the recommended physical activity levels, and, in general, with having better health outcomes, e.g., [12,29]. Based on Table 3, a higher percentage of white residents within the county is associated with an increased likelihood of having asthma. Considering this result in conjunction with the statistically insignificant coefficient estimate of the *Race* variable (which represents the race of the respondents) in the *Asthma diagnosis* model, the evidence on the role of race in asthma diagnoses can be considered inconclusive. Thus, further research may be required to clarify the link between race and asthma.

#### 3.3.2. Socioeconomic Attributes

The average marginal effects computed for the *Median annual household income* variable (Table 3) show that higher median income levels within the county are correlated with lower probabilities of being diagnosed with asthma and/or diabetes. Past research also found that increased income is associated with lower risk of asthma [21]. However, the direction of the effect of this variable in the *Diabetes diagnosis* model is not consistent with findings of Barr et al. [62], who suggested that higher household income levels were associated with a higher risk of diabetes.

As measures of a metropolitan area’s economy, the results for the *Average percentage of low-wage workers* and the *Average gross regional product* variables indicate that higher socioeconomic status and a larger-size economy within a metropolitan area are associated with a lower likelihood of residents being overweight or obese and having been diagnosed with asthma, as well as with a higher likelihood of residents participating in at least 150 min of physical activity per week and having a good or excellent general health status. These results are consistent with past findings, suggesting that: (*i*) higher incomes are associated with higher levels of physical activity, e.g., [29,63], and (*ii*) the likelihood of obesity and other adverse health outcomes declines with higher income levels and living in higher-income areas [15,21,29,36].

In addition, the coefficient (Table 2) and the average marginal effect (Table 3) of the *Average percentage of households with no cars* variable, which measures another aspect of the socioeconomic status (i.e., vehicle ownership levels) of residents of a metropolitan area, indicate that lower levels of vehicle ownership within metropolitan areas are associated with a lower likelihood of residents having good or excellent general health. One explanation for this result can be the limited access to healthcare providers or services, imposed by not having access to a car. Individuals who do not own a private vehicle may not be able to reach a physician’s office or pharmacy when they are ill and need treatment. As a result, they may be more likely to suffer from a poorer general health status.

Together, these results suggest that higher socioeconomic status within an area is associated with better health outcomes for residents, which is a reasonable finding. Higher socioeconomic status (e.g., higher incomes, owning a vehicle) means higher levels of affordability for better quality goods as well as higher levels of access to services that can impact one’s health. For example, high-income individuals can afford organically grown food items, better health insurance, and gym membership—all of which may favorably influence their health status. Past research found higher incomes to be correlated with better general health status [19,29,31,36,66]. The findings of the present study are also in line with arguments by Ellaway et al. [91], who suggested that vehicle ownership was associated with better health outcomes (i.e., lower rates of overall mortality and long-term illness, fewer symptoms, and gaining more psychosocial benefits).

#### 3.3.3. Crime-Related Attributes

Based on the coefficient estimates and average marginal effects of the *Average crime rate* variable, it seems that increased rates of violent crime within the metropolitan area are associated with lower probabilities of residents meeting the CDC’s recommendations on physical activity levels and having a good or excellent general health status, as well as with lower probabilities of them having been diagnosed with asthma. One explanation for these findings can be lower levels of outdoor physical activity due to safety concerns. Residents who fear violent crimes in their cities are not likely to spend much time outside of their houses to perform physical activities such as walking or bicycling. Fear and concerns about neighborhood crime and personal safety have been postulated to act as a barrier to walking and other physical activities [63,92], and empirical research has shown that violent crime rates have a negative impact on walking trips [33]. Further, chronic exposure to community violence and crime has been argued to be an important social environment factor that can potentially impact physical activity levels [40]. The latter study also suggested that the extent of environmental stressors (such as crime) may lead to reduced levels of individuals’ engagement in outdoor recreational physical activities (e.g., walking, bicycling, and use of open spaces for sports) [40].

On the other hand, residents of high-crime cities may be at lower risk of developing asthma due to spending more time indoors and having less exposure to airborne pollutants that are detrimental to respiratory health. Results also show a positive and significant association between the coefficient estimate (Table 2) of the *Average crime rate* variable and the probability of residents being overweight or obese and having been diagnosed with diabetes; however, the corresponding average marginal effects (Table 3) are not statistically significant, indicating the need for further investigation of the relationship between metropolitan area-level crime rates and obesity as well as diabetes rates in residents. Nonetheless, the positive direction of the effect of the *Average crime rate* variable in the *Overweight or obese* model (Table 2) is consistent with past findings suggesting that the prevalence of obesity is higher in areas with more violent crime [29], which lends a degree of confidence to the results obtained in the present study.

### 3.4. Travel Behavior Variables: Findings

As with the environmental factors, the effects of travel behavior-related factors on individuals’ health outcomes have been measured at two spatial levels: the county level and the metropolitan area level. These travel behavior variables characterize each Florida county and/or metropolitan area based on the level of usage of various modes of travel including walking and bicycling, private vehicle, and public transit. Measures of other travel behavior such as telecommuting and teleshopping have also been included in the analysis. The results of the models with regards to the travel behavior variables are discussed below.

#### 3.4.1. Active Travel

Table 2 and Table 3 indicate that the *Active travel mode share* variable, which represents the walking and bicycling travel mode share within a county, is negatively associated with the likelihood of being overweight or obese and positively associated with the likelihood of having good or excellent general health. Moreover, the results on the *Average walking and bicycling density* variable indicate that increased densities of active travel within a metropolitan area are associated with better health outcomes (i.e., a lower likelihood of asthma and/or diabetes diagnoses and a higher likelihood of having a good or excellent general health status). These results are in line with the large body of empirical research that points to a strong association between higher levels of active travel and improved health indicators, e.g., [8,9,13,14,15,16]. Thus, the findings of the present study corroborate past findings and provide further evidence that higher rates of walking and bicycling can lead to better health outcomes.

#### 3.4.2. Private Vehicle Travel

The results indicate that more traveling within the county by means of private vehicle (represented by the *Private vehicle travel mode share* variable) is associated with adverse health outcomes for residents, including a higher likelihood of having been diagnosed with asthma and/or diabetes (the average marginal effect is statistically insignificant in the case of diabetes). The result on asthma is reasonable and may be reflecting the effect of increased vehicle emission levels (due to increased use of automobiles within the county) on the respiratory health of residents. This result is in line with the literature suggesting that higher rates of automobile use and the resultant higher levels of vehicle emissions may lead to lung diseases such as asthma [71,74].

The adverse health effects of increased usage of private vehicles are also captured by the results on the *Average commuter stress index* variable, which represents the congestion-related stress endured by commuters within large urban areas [93]. This variable is positively associated with the probability of being overweight or obese (Table 3) and negatively associated with the probability of having a good or excellent general health status (Table 2 and Table 3). These results are consistent with past studies that found a link between increased use of automobiles and increased rates of obesity and/or a poor general health status, e.g., [8,20,21]. The results imply that, as expected, residing in metropolitan areas with higher levels of automobile-related commuter stress may adversely affect health outcomes. Lending further credence to this argument is past research suggesting that commute-related stress may be linked to adverse health outcomes, e.g., [23,75,89,94].

Together, these findings indicate that higher levels of private vehicle usage within the area of residence are associated with undesirable health outcomes, including higher levels of obesity and asthma, as well as a poorer general health status for residents.

#### 3.4.3. Public Transit Travel

The coefficient estimates (Table 2) and average marginal effects (Table 3) of the *Public transit mode share* variable are associated with a few favorable health outcomes such as a lower probability of being overweight or obese, a lower probability of having been diagnosed with diabetes, and a higher probability of meeting the CDC recommendations on physical activity levels. However, this variable is also associated with unfavorable health outcomes including a higher probability of having been diagnosed with asthma and a lower probability of having a good or excellent general health status.

These findings are consistent with the literature suggesting that: (*i*) a positive association exists between public transit use and physical activity in the form of active travel, as using public transit involves walking (and/or bicycling) to, from, and within the transit stations, e.g., [9,27,62,67,95,96]; and (*ii*) public transit use is correlated with a lowered risk of being overweight or obese due to the active travel involved in transit trips, e.g., [19,67]. The results are also consistent with those of past studies that found an association between use of subway and lower probabilities of obesity and diabetes [15], as well as those that reported an association between use of public transportation and better weight-based health outcomes, including lower risks of being overweight or obese [9,20,67].

With respect to general health, however, the results of the present study stand in contrast with those of a few past studies that found that higher levels of transit use within a county were correlated with better general health for residents [20,21]. One explanation for the adverse effects of increased transit use within an area on general health status of residents can be conditions that are typically associated with public transit use and have a potential to affect human health. For transit users, these could include long wait times, higher stress levels due to unpredictability of service, exposure to higher levels of air pollution due to transit vehicle operations, exposure to inclement weather conditions, exposure to crowded stations, and interaction with other users who may be ill. Past studies suggest that public transit users experience higher levels of perceived stress, particularly due to factors such as long commutes and unpredictability [88,89]. The chronic stress experienced by public transit users may lead to adverse health outcomes and, consequently, to a poor general health status. In addition, the general health of transit users may be adversely affected due to increased and frequent exposure to other riders and higher disease diffusion rates among riders, particularly for airborne infectious diseases such as the common cold. Support for these arguments is provided by a few previous studies, which suggested that: (*i*) densely crowded and often poorly ventilated environments associated with public transit can provide high respiratory contact rates and thus pose a risk of transmission of airborne infections [97]; and (*ii*) crowded transit vehicles provide increased opportunities for diseases to spread, which can negatively affect individuals’ personal health [22].

Moreover, and for both users and non-users of transit, the higher air pollution levels due to the increased transit use—particularly in the case of bus or other vehicular transit—within the county may lead to negative health outcomes. Past research suggests that air pollution from vehicles has negative health effects including lung disease and other undesirable health conditions [71]. With regards to asthma, additional amounts of walking or waiting by transit users may negatively affect their respiratory health and lead to asthma due to inhalation of more polluted air. These arguments are supported by findings of Samimi and Mohammadian [21], who found that increased transit use within a county was correlated with higher rates of asthma. They suggested that this was due to transit riders being more exposed to polluted air because of the additional walking associated with transit use. Nonetheless, non-transit users who live in areas within which transit use is high can also be at increased risk of developing asthma due to the higher air pollution levels within their residential areas.

Further, the results presented in Table 2 and Table 3 indicate that increased public transit usage within the metropolitan area (represented by the *Annual public transportation passenger-miles* variable) is associated with a lower likelihood of being overweight or obese and a lower likelihood of having been diagnosed with diabetes. However, this variable is also associated with a higher likelihood of asthma diagnoses and a lower likelihood of having a good or excellent general health status. These effects are in line with those at the county level. Cumulatively, these results suggest that despite having a potential to promote physical activity and provide some health benefits, increased transit use within the area of residence may also have a few health-related drawbacks, including increased rates of asthma and poorer general health status for residents.

#### 3.4.4. Telecommuting

The estimation results on the variable representing the level of telecommuting within a county (i.e., the *Average frequency of telecommuting events per month* variable) indicate that higher rates of telecommuting within a county are associated with a higher likelihood of being overweight or obese (Table 3), a lower likelihood of meeting the CDC recommendations on physical activity levels, and a lower likelihood of having a good or excellent general health status (Table 2 and Table 3). Increased rates of telecommuting within a county are also associated with a lower likelihood of being diagnosed with asthma for residents (Table 2 and Table 3). The results further suggest that a negative association exists between the *Average percentage of household members with telecommuting option* variable and the likelihood of being diagnosed with asthma as well as the likelihood of meeting the CDC’s recommendations on physical activity levels (Table 2 and Table 3). The results show consistency in terms of the direction of associations between the two measures of telecommuting behavior of a county’s population and their health outcomes.

These findings provide evidence for arguments presented in previous research that excessive participation in computer-related activities has a potential to reduce physical activity levels [40]. More telecommuting can mean less physical activity, which can lead to obesity and other adverse health outcomes. In addition, facilitated access to food for telecommuters who work from the convenience of their homes can lead to an increased overall level of food consumption and, ultimately, to obesity and other adverse general health outcomes. Nonetheless, with regards to obesity and physical activity levels, the results of the present study are not consistent with those of Henke et al. [39], who found that non-telecommuters were at greater risk for obesity and physical inactivity, and those of Tajalli and Hajbabaie [15], who did not find a statistically significant association between telecommuting and obesity. One reason for the inconsistency in results can be that in both of the referenced studies [15,39], telecommuting behavior information was available at the respondent level, which made it possible to examine the association between telecommuting behavior and health outcomes at the individual level. Telecommuting data at the respondent level were not available for the 2009 BRFSS database, which is the person-level health database used in the present study; therefore, this analysis examined the role of telecommuting in health outcomes using aggregate telecommuting measures (i.e., county-level measures) derived from the 2009 NHTS data. Caution should thus be exercised with respect to the findings of the present study, bearing in mind the issue of *ecological fallacy*, which is defined as deducing inferences about correlations at the disaggregate level when data are only available at the aggregate level [98,99]. Nevertheless, the inconsistency in the results of the present study and past findings suggests that further investigation into the relationship between telecommuting and health outcomes may be needed, particularly in the case of obesity and physical activity.

The negative association between telecommuting measures and the likelihood of asthma diagnoses is notable. Increased levels of telecommuting within an area may mean less vehicular commute and less congestion, which can lead to reduced air pollution levels in that area. Past research suggests that telecommuting has a potential to serve as a substitute for commuting and can thereby reduce traffic congestion as well as greenhouse gases and improve air quality in urban areas [38,100,101]. The cleaner air within the area of residence due to higher telecommuting rates can then lead to a lower risk of asthma for residents.

With regards to diabetes, the average marginal effects (Table 3) for the measures of telecommuting in the *Diabetes diagnosis* model are statistically insignificant. This result is consistent with past empirical research that did not find a statistically significant link between telecommuting and diabetes [15].

#### 3.4.5. Teleshopping

The results also provide evidence that the extent of online shopping-related activities within an area is associated with the health status of residents of that area. Most notably, the model estimates (Table 2) and average marginal effects (Table 3) of the *Average number of online purchases per month* and the *Average number of monthly deliveries related to online purchases* variables indicate that increased online shopping-related activities are associated with a lower likelihood of meeting the CDC’s recommendation on physical activity and a higher probability of being overweight or obese. These results complement those obtained on the telecommuting-related variables and are consistent with them in the direction of correlations. The findings are also in line with past arguments that excessive participation in computer-related activities and time spent online have a potential to reduce physical activity levels [40,41] and may contribute to weight gain [41]. Online shopping can be considered a feature of a sedentary lifestyle. Teleshoppers do not need to leave their house (or even their couch) to satisfy their shopping needs. Therefore, it is plausible to assume that if performed habitually, online shopping may lead to lower levels of physical activity and, ultimately, to adverse health outcomes such as obesity. Considering these arguments, it is not surprising to see that increased levels of online shopping within a county are also correlated with a lower likelihood of having a good or excellent general health status for the residents (Table 2 and Table 3). In a general internet overuse context, these results are consistent with those of Zheng et al. [41], who found increased frequencies of online activities were strongly associated with higher levels of complaints related to general physical health outcomes.

The only favorable online shopping-related results are those in the *Asthma diagnosis* model. Table 2 and Table 3 indicate that increased levels of online shopping-related activities within a county are associated with a lower likelihood of residents being diagnosed with asthma. This finding may be related to the substitution effects of online shopping. Online shopping has a potential to substitute for some of the vehicular trips that individuals would otherwise make to stores to satisfy their shopping needs. For instance, some studies argue that online shopping and home delivery provide the opportunity to reduce the total vehicular transportation related to grocery shopping and the associated emission levels [102,103]. Thus, the negative association between online shopping-related variables and asthma diagnosis may be due to the reduced number of vehicular trips to stores and the associated lower air pollution levels within the county of residence.

Nonetheless, literature related specifically to health impacts of online shopping is scarce, and little empirical knowledge exists in this area—making it difficult to compare findings from the present analysis to those of past studies. The results of the present study fill that gap in empirical research and suggest that online shopping may contribute to adverse outcomes (except in the case of asthma)—a finding that is open to future and further research.

### 3.5. Endogeneity and Reverse Causality between Health Outcomes and Physical Activity

An underlying assumption in developing the person-level health outcome models for this study was that the *Physical activity minutes* variable, which represents the individual’s total minutes of moderate physical activity per week, is an endogenous independent variable in the models. This means that after controlling for all the other independent variables, there is a non-zero correlation between the *Physical activity minutes* variable and the error term in the models. This correlation could exist due to reverse causality or the effect of omitted variables (see Section 2.5).

The Wald test of exogeneity of the instrumented variable (i.e., *Physical activity minutes* variable) shows significant results in the *Overweight or obese*, *Asthma diagnosis*, and *Diabetes diagnosis* models (see Table 2). This indicates that the null hypothesis of no endogeneity can be rejected in these models and the employment of an instrumental variable (IV) binary probit model is justified to control for endogeneity bias in the models.

Moreover, the results of the Amemiya-Lee-Newey minimum χ^2^ test for validity of instruments are statistically insignificant for the equivalent *Overweight or obese*, *Asthma diagnosis*, and *Diabetes diagnosis* models estimated using the twostep method see [61]. This indicates that the selected instrumental variables (i.e., the *Employment status*, *College education*, and *Children* variables) are valid instruments for the endogenous physical activity independent variable (i.e., the *Physical activity minutes* instrumented variable) in the IV binary probit models presented in this study.

Findings of past research lend some degree of confidence to the results of the test for validity of instruments. With respect to educational attainment, some studies suggested that higher education had: (*i*) a negative correlation with BMI [12,29,60]; (*ii*) a negative correlation with the probability of being obese [8,15,21,29]; and (*iii*) a negative correlation with the probability of having diabetes [15,29]. Conversely, others found either a positive association between higher education and the probability of obesity and diabetes [62] or no significant correlation between individuals’ educational status and obesity or other health outcomes [19]. Further, Braun and Malizia [66] found that although an increased proportion of the county population with higher educational attainment was not associated with prevalence of obesity within the county, it was correlated with lower prevalence of diabetes. These findings suggest that there is no consensus in past research regarding the association between educational status and obesity or diabetes. It should also be borne in mind that none of these studies controlled for endogeneity bias in their analysis; therefore, any correlations observed between education and obesity or diabetes could be due to the indirect effects of education on these health outcomes through impacting physical activity levels—a condition that enhances the validity of educational attainment as an instrument for physical activity in modeling obesity and diabetes. Moreover, past research has not found a correlation between higher education and having asthma [19,21]. The inconsistencies in the statistical significance and direction of the correlations between educational attainment and obesity, diabetes, and asthma can mean that education is not related to these health outcomes—at least directly. On the other hand, research provides evidence that higher education is associated with higher likelihood of participation in physical activity as well as higher levels of physical activity, e.g., [12,63,65]; therefore, educational attainment can be considered as a valid instrument for physical activity in modeling obesity, diabetes, and asthma—as performed in the present analysis.

The result of the current study, which finds the number of children living in the household as a valid instrument for physical activity in modeling obesity and asthma, can also be further validated based on past findings that found no direct correlation between presence of children in a household and health outcomes but reported observing a correlation between presence/number of children in a household and physical activity. Examples of these studies include those that found: (*i*) having children was not correlated with obesity or asthma [19,21]; but that (*ii*) having more children in the household was positively associated with physical activity in terms of active travel [104]; and (*iii*) being in a family with children was not a barrier to physical activity in the form of cycling [105].

## 4. Conclusions

Research thus far asserts that human health is affected by several factors including the travel behavior of individuals as well as the built and social environments of their surroundings. However, while many studies in the past examined the relationship between various health outcomes and built environment factors at the neighborhood and county level, little attention has been given to the health impacts of built environment characteristics at larger geographical scales such as those of the metropolitan area of residence. To narrow that knowledge gap, and considering the principles of the ecological model, the present study tested the hypothesis that built and social environments at two spatial levels (i.e., the county level and the metropolitan area level) influence individuals’ health outcomes. Measures of travel behavior at the county and metropolitan area levels were also included in the analysis to probe the role of travel behavior within communities—including the under-investigated role of telecommuting and teleshopping behaviors—in individuals’ health. The study conclusions are discussed below.

### 4.1. Research Findings

The analysis results provide evidence that in addition to personal and household characteristics, human health is affected by the characteristics of the built and social environments. These two aspects of the environment exert their influence on human health through various domains and at hierarchical spatial levels of influence, including the previously under-examined metropolitan area level.

The findings indicate that despite being associated with increased levels of physical activity, counties with higher levels of compactness may adversely affect the health outcomes of residents—potentially due to quintessential characteristics of dense urban areas such as crowded conditions, higher pollution levels, and increased stress levels. Counties with a higher extent of mixed-use development have a potential to promote physical activity but can also lead to some unfavorable health outcomes for residents, including a higher risk of asthma. On the other hand, poorer health outcomes are associated with living in sprawled counties where average distances to local transit stops are greater. Considering these findings, there may be an optimal threshold for counties to become dense and mixed (in terms of land use) to promote residents’ health.

Findings also indicate that increased transit accessibility to employment opportunities as well as increased pedestrian friendliness of the street network within the county can contribute to lower rates of obesity. Other county-level built environment attributes that can contribute to better health outcomes for residents are increased access to clinical healthcare, recreational facilities (e.g., parks), and healthy food outlets. In contrast, living in counties with higher pollution levels and higher access to unhealthy food outlets may lead to adverse health outcomes for residents.

A salient finding of this analysis is that the influence of a few built environment factors—including mixed-use development, intersection density, access to local transit, and accessibility to employment opportunities—on health extends beyond the county boundaries and into the metropolitan area. For instance, the findings provide evidence that metropolitan areas with increased intersection density (a proxy for compactness in terms of street network connectivity) may promote the general health of residents. Based on this finding, it can be inferred that a better general health status may be obtained by residing in a more compact, better connected metropolitan area than in a compact neighborhood or county located within a sparse metropolitan area—as also suggested by Marshall et al. [31]. The study findings further suggest that living in sprawled metropolitan areas with lower levels of access to local transit may lead to obesity and other adverse health outcomes. On the other hand, increased levels of regional transit accessibility to employment within the metropolitan area may promote physical activity and health of residents. Further, residents of metropolitan areas with more roadway congestion may suffer health consequences due partly to higher stress levels and lower physical activity levels associated with commuting on congested roadways. Considering these findings, it can be concluded that although the county-level built environment may be more influential in the health status of residents, the effects of the built environment factors at larger spatial scales such as the metropolitan area on individuals’ health are existent and significant and, therefore, should not be overlooked in analysis of health outcomes.

With respect to the social environment, the findings of the present study suggest that the median age, racial composition, and household income levels within the county of residence can affect residents’ health outcomes. Moreover, and as with the case for the built environment, the social environment’s influence on human health extends beyond the county boundaries. One example is the effect of the metropolitan area’s gross regional product (GRP), which is considered one of several measures of the size of the economy within a metropolitan area. The study findings provide evidence that metropolitan areas with a stronger and larger economy (i.e., higher average GRP) can promote residents’ health. In addition, residents of metropolitan areas within which income and car ownership levels are higher enjoy improved health outcomes. Other findings imply that residents of metropolitan areas with higher violent crime rates may suffer health-related implications, including lower levels of physical activity and a poorer general health status.

Further, the study findings show that living in counties or metropolitan areas where active travel is occurring at higher rates is associated with better health outcomes, including lower rates of obesity and a better general health status. On the other hand, residents of communities within which usage of private vehicles is more common and the levels of automobile-related commuter stress are high may suffer from adverse health outcomes. These findings are not surprising and corroborate the findings of past research asserting that the physical inactivity, the sedentary lifestyle, as well as the chronic stress related to operating a vehicle and car commuting contribute to a declined health status. In addition, residing in county and metropolitan areas where public transit usage is prevalent may help in lowering the risk of obesity and diabetes, but can also lead to a higher risk of asthma and a poorer general health status—presumably due to reasons such as increased exposure to polluted air generated from transit vehicles, increased exposure to harsh weather conditions, increased exposure to higher disease diffusion rates due to crowded vehicles, and increased levels of stress related to commuting by public transit. In terms of the role of telecommuting and teleshopping behaviors in health, the findings suggest that living in communities where telecommuting and teleshopping activities are prevalent among residents may lead to unfavorable health outcomes such as reduced levels of physical activity and increased rates of obesity.

The findings also imply that reverse causality exists between health outcomes and physical activity—leading to a potential endogeneity bias, which should not be overlooked in examining the links between physical activity and health outcomes. The capabilities of the instrumental variable (IV) analysis provide an appropriate tool for estimating such complex links within a comprehensive model framework, as performed in the present study.

**Figure 5:**
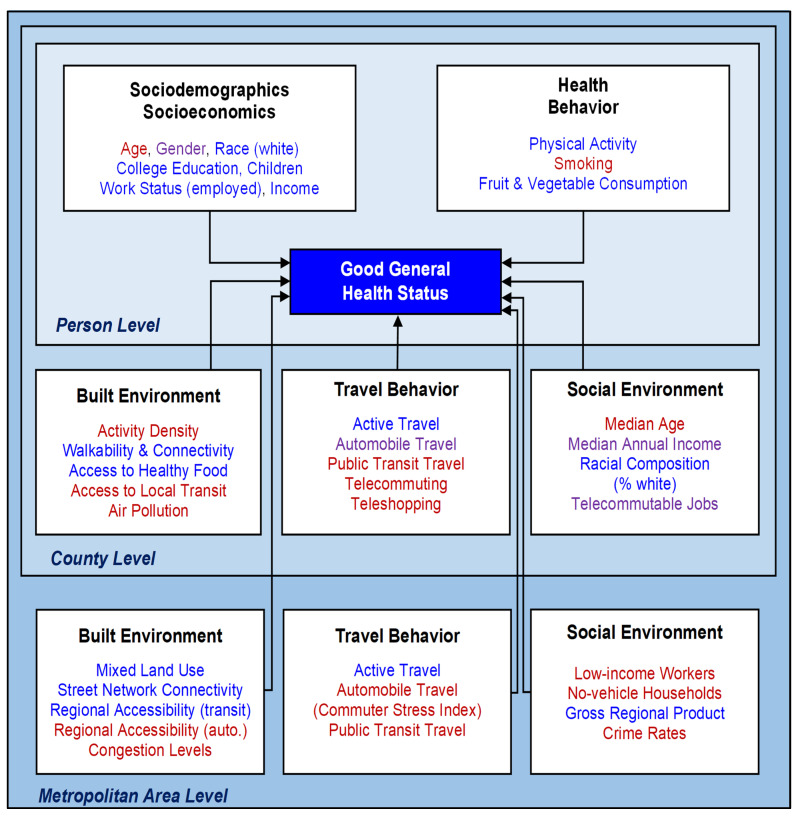
Infographic for summarized findings with regards to the respondents reporting a good general health status (Legend: Arrow indicates a correlation; blue font, positive correlation; red font, negative correlation; purple font, non-statistically significant correlation).

Figure 5 shows an infographic that summarizes the study findings based on the estimated average marginal effects (Table 3) and with respect to the roles of various factors in having good general health as a key indicator of individuals’ overall health status.

### 4.2. Research Contributions

This research contributes to the body of knowledge on the interrelationships between the environment (built and social), travel behavior, and health in terms of theoretical framework, empirical findings, and policy debates.

Regarding theoretical contributions, the study considers past research that emphasizes the role of multiple spatial scales of the environment in health outcomes [57] to derive a theoretical framework for disentangling the interlinks among built and social environments, travel behavior (e.g., active travel), and health. The ecological framework presented in this study allows for testing the health impacts of various dimensions of the built and social environments at multiple spatial levels (i.e., county and metropolitan area) simultaneously, thereby offering a comprehensive approach that has rarely been applied to empirical data.

In terms of empirical findings, the analysis provides evidence that the metropolitan area-level built environment attributes play an important role in residents’ health outcomes. As such, the present study contributes by strengthening and complementing the existing empirical knowledge on the role of the built environment in human health (which in the existing literature is mainly focused on the role of neighborhood- and/or county-level built environment attributes in health). Additionally, this research contributes to the body of empirical knowledge on the link between travel behavior and health by probing the roles of telecommuting and teleshopping behaviors in health outcomes. More specifically, the study findings shed light on the role of telecommuting behavior in physical health outcomes, as very little empirical evidence exists on that topic. Further, empirical studies with respect to health impacts of teleshopping are—to the best of authors’ knowledge—nonexistent. Thus, this study contributes by filling that gap in research by empirically testing the link between measures of teleshopping behavior and health outcomes.

In terms of contributions to policy and practice, the study findings contribute to the ongoing policy debates concerning the role of the built environment in physical activity and health outcomes. As this research investigates the health impacts of the built and social environment attributes at the two hierarchical levels of county and the metropolitan area, the research findings particularly shed light on the most promising policy interventions that can improve public health through modifications to the built and social environments in urban areas. This will enable transportation planning, urban design, and public health decision-makers to develop more effective policies that optimize the efficiency of resource usage as well as the societal health benefits.

### 4.3. Policy Implications

Considering the built environment, the findings of this study imply that policies concentrating on interventions that target the built environment of the county may be effective in promoting essential health provisions. These interventions include modifications to the built environment of the county that make it more conducive to physical activity and active travel. Other interventions include modifications to the built environment of the county to make it more promotive of healthy food choices (or as described by Joshu et al. [57], to make it more “non-obesogenic”). Examples of such interventions are changes to the built environment features throughout the county that:

- Increase walkability and pedestrian friendliness of the street network;
- Increase connectivity of the street network (to support active travel);
- Facilitate access to healthy food outlets;
- Facilitate access to parks, green spaces, and recreational facilities;
- Facilitate access to clinical healthcare;
- Limit the number of fast food restaurants; and
- Lower ambient air pollution levels.

The built environment characteristics of the metropolitan area also prove to be influential in individuals’ health outcomes; therefore, these characteristics should also be considered in policy and health intervention decision-making processes. Based on the findings of the present study, more effective public health policies and interventions seem to be the ones that consider the overall form of the metropolitan area in addition to that of the county. These include interventions that can modify the built environment within the entire metropolitan area in such a way to:

- Increase compactness (in terms of intersection density and street connectivity);
- Increase access to transit (e.g., shorter distance to transit stops);
- Lower roadway congestion levels and commute durations.

As an example, constructing new compact neighborhoods with high intersection densities in the middle of sprawled suburban areas may not provide optimal public health benefits. Instead, it is the overall character of the metropolitan area that is more influential. Residing in a connected neighborhood or county can potentially yield more health benefits in a metropolitan area with a connected street network structure than in a metropolitan area with a disconnected street network. Thus, city planning strategies and urban design policies aiming at building compact metropolitan areas with better connected street networks can promote public health.

With regards to social environment, the findings imply that health-promoting policies for urban areas include those that:

- Increase the size and strength of the economy (e.g., a higher GRP); and
- Lower violent crime rates.

Considering the case of crime rates as an example, interventions that help reduce fear-producing behavior and promote crime-related safety can potentially improve the health of residents within an urban area. Such interventions can include: improving street lighting; improving lighting as well as visibility from surrounding buildings and stores at transit stations, bus stops, and parking lots; reducing the number of vacant lots and dilapidated buildings as well as providing emergency pedestal phones and call boxes along walking and bicycling pathways in parks. Another important strategy to lower crime rates within cities can be increasing surveillance or “number of eyes” on streets [106] by promoting urban designs that encourage continuous presence of people on city streets. In that respect, mixed-use developments can be effective. Presence of various establishments such as restaurants, stores, and other public places that are open during later hours of the evening can generate constant presence of people, which by increasing the number of eyes and ears, can help in monitoring the street and reducing the number of crimes. However, as findings of this study indicate, an optimal threshold may exist for cities to become mixed in terms of land use and still remain beneficial to public health. Therefore, urban design policies aiming at increasing the extent of mixed-use development within metropolitan areas should be developed based on optimization of such trade-offs.

Further, based on the study findings, policies that encourage travel by active modes (i.e., walking and bicycling) and the public transit mode may be cost-effective interventions in increasing physical activity and preventing obesity and can thereby promote public health for residents of urban areas. This is crucial information for transportation planning and public health policymakers seeking to improve public health through changes to individuals’ travel behavior. The conclusion of this study regarding the benefits of promoting active travel (through changes to the built and/or social environments or other policies such as educational programs), albeit not surprising, reaffirms past research conclusions that environments supportive of active travel promote individuals’ health status. In addition, since using public transit involves active travel at both ends of a trip, promoting a higher public transit mode share can also serve as an effective policy to improve some public health outcomes by integrating physical activity into the daily routines of individuals.

Overall, evidence found in this study supports the notion that adverse health outcomes can be ameliorated through interventions that target the built and social environments as well as the travel behavior of individuals. The analysis framework and findings presented in this research can assist policy decision-makers in assessment of built and social environment attributes—at multiple spatial levels—as well as the travel behavior trends within communities that have a potential to influence health outcomes of residents. This, in turn, can help in making more informed decisions and developing more effective policies based on interventions that would yield the most efficient use of resources and the greatest health benefits. Such policies can improve individuals’ health conditions in various ways and lead to a better state of health for communities and the society as a whole. The annual societal healthcare cost of lack of physical activity and its resultant negative health outcomes in the U.S. is estimated to be around 117 billion dollars [1]. Bearing that in mind, the conclusions of this study can be helpful in creating policies that spatially optimize modifications to the built and social environments and thereby reduce the burden of healthcare costs on society.

### 4.4. Study Limitations and Future Research

The current study has a few limitations. First, due to lack of a database that provided concurrent data on travel behavior, the built environment, and health outcomes, several databases were linked to obtain a combined dataset for this analysis. Additionally, due to privacy issues with health data, travel behavior data were aggregated to the county level—which may have subjected the study findings to ecological fallacy. Using a comprehensive database that provides health status as well as travel behavior information at the person level and location data (preferably at the neighborhood level) can enhance analysis of the links between individuals’ health, their travel behavior, and the built and social environment attributes of their residential area. Such a database, however, remains scarce—limiting the boundaries of research. Ideally, national travel and health surveys can be modified in the future in such a way to provide data on travel behavior, health outcomes, and the built environment in one database. This will also allow compilation of a rich inventory of travel behavior, health, and built environment data over time, which can facilitate future longitudinal analysis of the interlinks between the three.

Another data-related limitation was the incomplete consideration of transportation-specific physical activity in the health database that was used in this analysis (i.e., 2009 BRFSS). This was owing to the BRFSS survey: (*i*) not distinguishing between active travel and other forms of physical activity; and (*ii*) not including the amount of work-related active travel in its questionnaire. Concerning the first issue, it should be borne in mind that moderate physical activity has been defined in 2009 BRFSS as “brisk walking, bicycling, vacuuming, gardening, or anything else that causes some increase in breathing or heart rate” [55]. As a result, the specific amount of walking/bicycling (i.e., active travel) by each respondent is not clear, as it is combined with other physical activities that the respondent might have engaged in. Regarding the second issue, and as also noted in previous research [27], it seems that the BRFSS is mainly focused on leisure-time physical activity, which results in a lack of data on the amounts of work-related active travel. Nonetheless, surveys such as the BRFSS are the most promising sources of physical activity and health data that can be linked to built environment factors [107]. Thus, future research on the interlinks among physical activity, the built environment, and health can greatly benefit from refined versions of such surveys that allow recordation of exercise and utilitarian active travel separately from each other, and also separately from other forms of leisure-time physical activity.

In addition, the health data used for developing the health outcome models in this study came only from metropolitan areas within the state of Florida. Future research can incorporate data from additional metropolitan areas in other U.S. states (or other countries) in probing the health impacts of travel behavior including active travel, telecommuting, and teleshopping behaviors as well as those of the built and social environments at various spatial levels. Such an enhanced database will allow better examination of the effects of various measures of travel behavior and various spatial scales of the environment (i.e., built and social) on individuals’ health. Nonetheless, while the metropolitan areas selected for this study are not perfectly representative of the U.S. metropolitan areas (or urban areas within any other country), the analysis framework offers a basis for examining the health status of residents of different urban areas based on differences in their travel behavior and the environmental attributes of their residential location. Future analysis based on data from additional metropolitan areas in the U.S. (or other counties) can also provide insights into the generalizability of the findings of the present study.

Further, as in many previous studies, a major limitation of the current study is the usage of cross-sectional data. The employment of instrumental variable (IV) techniques—to some extent—allowed for examination of reverse causality between physical activity and health outcomes. Nonetheless, the use of cross-sectional survey data is a practical drawback to these techniques, limiting the ability to make true causal inferences. It should further be noted that the link between health outcomes and the built environment may also be bidirectional, as healthier people may self-select themselves into health-promoting residential areas. The bidirectional effects between health outcomes and the built environment were not accounted for in this study, which can be an avenue of research in subsequent work.

In addition, the effects of several other built environment factors on health outcomes of individuals were not considered in this study and can be examined in future research. These include, but are not limited to, availability and cost of parking and existence as well as extent of traffic calming measures within the residential area.

Another topic that was not addressed in this study is the uncertain geographic context problem, which deals with the potential uncertainties that exist with respect to individuals’ exposure to environmental (i.e., contextual) influences when measuring their health outcomes. According to Kwan [108], these uncertainties include: (*i*) the spatial uncertainty in the actual areas that exert the contextual influences on health outcomes, and (*ii*) the temporal uncertainty in the timing and duration for which individuals were exposed to certain health-affecting contextual influences. Contextual influences can vary over space and time in a complex way, which can greatly complicate examination of their effects on health [108]; thus, the role of *human mobility* in measuring people’s health outcomes is emphasized through the uncertain geographic context problem. Emerging sources of data, such as mobile-device big data, which can assist in measuring human mobility and the population’s exposure to contextual (both built and social environment) influences, provide an opportunity for future research on the roles of travel behavior, human mobility, and environment in health. For instance, earlier research has shown that using big data to integrate the dynamics of human mobility, population distribution, and geographic locations of urban greenspace into the exposure assessment is a more reasonable method to assess population exposure to urban greenspace—a built environment attribute with a potential to improve individuals’ physical and mental health [109].

Moreover, other important health indicators such as high blood pressure, heart disease, stroke, and safety-related health outcomes (e.g., injuries and fatalities from crashes) were not considered in this study. Future research can use the ecological framework presented in this study to examine the health effects of various measures of travel behavior (including telecommuting and teleshopping) as well as various measures of the built and social environments at multiple spatial levels.

Lastly, notably absent among the factors included in this analysis were biological susceptibility factors. While there is an underlying genetic basis for health status of individuals and their levels of susceptibility to disease, the role of genetic predispositions in an individual’s health outcomes was not controlled for in the health models presented in this study. The reason for this was unavailability of data on biological factors. An individual’s health outcomes, including his/her physical activity levels are, nevertheless, dependent on biological factors and heredity, e.g., [29,58,64]. Thus, future research can benefit from inclusion of genetic factors in the analysis to disentangle the interlinks between travel behavior, physical activity levels, built and social environment factors, and health outcomes.

## Figures and Tables

**Table 1:** Health outcome models’ variables.

Model Variables	Mean	Std. Dev.	Data Source
***Dependent Variables* (*Individual-level Health Outcomes*)**			
Overweight or obese (individual had a BMI ≥ 25—1: yes, 0: no)	0.63	0.48	BRFSS ^1^
Diabetes (individual was diagnosed with diabetes—1: yes, 0: no)	0.14	0.34	BRFSS ^1^
Asthma (individual was diagnosed with asthma—1: yes, 0: no)	0.12	0.32	BRFSS ^1^
Good general health (individual reported a “good or better” health status—1: yes, 0: no)	0.78	0.41	BRFSS ^1^
CDC physical activity (individual participated in 150 min of physical activity/week per CDC guidelines—1: yes, 0: no)	0.62	0.49	BRFSS ^1^
***Independent Variables***			
**Individual and Household Attributes**			
Age (individual’s age in years)	58.98	16.27	BRFSS ^1^
Race (individual’s race:—1: white, 0: otherwise)	0.82	0.38	BRFSS ^1^
Gender (individual’s gender:—1: male, 0: female)	0.38	0.49	BRFSS ^1^
Employment status (individual was employed—1: yes, 0: no)	0.42	0.49	BRFSS ^1^
College education (individual had a college degree—1: yes, 0: no)	0.61	0.48	BRFSS ^1^
Physical activity minutes (individual’s total minutes of moderate physical activity per week) ^2^	58.79	82.21	BRFSS ^1^
Fruit and vegetable consumption (individual’s fruit and vegetable servings per day)	3.96	3.04	BRFSS ^1^
Smoking status (1: everyday smoker, 2: someday smoker, 3: former smoker, 4: non-smoker)	3.23	1.09	BRFSS ^1^
Drinking status (total number of alcoholic beverages consumed per month)	11.92	41.34	BRFSS ^1^
Household’s income category ^3^	3.48	1.44	BRFSS ^1^
Number of children in household (number of children under 18 years of age in individual’s household)	0.42	0.92	BRFSS ^1^
**Built Environment Attributes**			
**County Level**			
Mean activity density [average (employment + housing units)/acre)]	3.41	2.99	SLD ^4^
Mean entropy (average 5-tier employment entropy)	0.54	0.07	SLD ^4^
Mean intersection density [average (automobile-oriented intersections/mi^2^)]	0.72	0.55	SLD ^4^
Mean local transit accessibility [average (distance to the nearest transit stop in meters)]	735.45	69.64	SLD ^4^
Mean temporal automobile accessibility (average number of jobs within a 45 min automobile commute)	44,173	46,063	SLD ^4^
Mean temporal transit accessibility (average number of jobs within a 45 min transit commute)	1488	1666	SLD ^4^
Density of fast food restaurants ^5^ (number of fast food restaurants/10,000 population)	1.97	0.63	POI ^6^ and ACS ^7^
Access to parks (percentage of population living within half-mile of park features) (%)	20.54	15.21	CHSI ^7^
Primary care physician rate (primary care providers per 100,000 population)	68.10	31.11	CHR&R ^7^
Access to healthy food outlets ^8^ (percentage of zip codes with healthy food outlets) (%)	47.68	11.21	CHR&R ^7^
Ambient air pollution (annual number of unhealthy air quality days due to ozone and fine particulate matter)	7.23	4.89	CHR&R ^7^
Mean Walk Score (dimensionless)	11.23	18.94	Walk Score^®^
**Metropolitan Area Level**			
Mean activity density [average (employment + housing units)/acre)]	4.39	2.40	SLD ^4^
Mean entropy (average 5-tier employment entropy)	0.56	0.05	SLD ^4^
Mean intersection density [average (automobile-oriented intersections/mi^2^)]	0.88	0.42	SLD ^4^
Mean local transit accessibility [average (distance to the nearest transit stop in meters)]	669.51	46.33	SLD ^4^
Mean temporal automobile accessibility (average number of jobs within a 45 min automobile commute)	53,611	40,868	SLD ^4^
Mean temporal transit accessibility (average number of jobs within a 45 min transit commute)	1,703	1,450	SLD ^4^
Mean roadway congestion index (dimensionless)	0.98	0.18	UMI ^7^
**Social Environment Attributes**			
**County Level**			
Median age (years)	39.19	4.85	ACS ^7^
Median annual household income (dollars)	43,842	6292	ACS ^7^
Percentage of white population (%)	76.70	7.59	ACS ^7^
Percentage of telecommutable jobs ^9^ (%)	56.40	5.82	CEDDS ^10^
**Metropolitan Area Level**			
Average percentage of low-wage workers (workers earning ≤ USD 1250/month) (%)	27.44	1.93	SLD ^4^
Average percentage of households with no cars (%)	6.43	1.19	SLD ^4^
Average gross regional product (GRP) (in millions of 2004 U.S. dollars)	49,829	63,022	CEDDS ^10^
Average crime rate (annual violent crimes/100,000 population)	700.83	363.17	UCR and CHR&R ^7^
**Travel Behavior Attributes**			
**County Level**			
Active travel (i.e., walking and bicycling) mode share (%)	8.10	2.38	ACS ^7^
Private vehicle travel mode share (%)	87.16	3.60	ACS ^7^
Public transit travel mode share (%)	1.21	0.79	ACS ^7^
Average frequency of telecommuting events per month	4.05	2.03	NHTS ^11^
Average percentage of household members with telecommuting option (%)	11.46	4.83	NHTS ^11^
Average number of online purchases per month	1.80	0.37	NHTS ^11^
Average number of monthly deliveries related to online purchases	3.00	0.48	NHTS ^11^
**Metropolitan Area Level**			
Average walking and bicycling density [average (number of walking and bicycling trips in CBG/CBG area in acres)]	0.0024	0.0016	NHTS ^11^ and SLD ^4^
Annual public transportation passenger-miles (millions)	129.14	262.00	UMI ^7^
Average commuter stress index	1.18	0.064	UMI ^7^
**Number of Observations (i.e., BRFSS Respondents) = 9427** **Number of Counties = 51; Number of Metropolitan Areas = 23**			

^1^ This study uses the 2009 BRFSS dataset; ^2^ BRFSS defines moderate physical activity as “brisk walking, bicycling, vacuuming, gardening, or anything else that causes some increase in breathing or heart rate” [55]. ^3^ The household income categories in BRFSS are: 1: < $15,000; 2: $15,000 to < $25,000; 3: $25,000 to < $35,000; 4: $35,000 to < $50,000 and 5: ≥ $50,000) [55]. ^4^ This study uses data from SLD version 2.0, which was released in 2013, but uses 2010 Census data [56]. ^5^ The following establishments have been considered fast food restaurants in this study: Burger King, Domino Pizza, KFC, McDonalds, Papa John’s Pizza, Pizza Hut, Roy Rogers, Subway, Taco Bell, and Wendy’s. ^6^ This study uses 2011 POI data. ^7^ This dataset provides multiyear data; thus, multiyear averages have been used in this study. ^8^ According to the CHR&R dataset, healthy food outlets include grocery stores and produce stands/farmers’ markets. ^9^ Percentage of county jobs that fall into the “Information”, “FIRE” (Finance, Insurance, Real Estate), “Services”, and “Government” sectors. ^10^ Source: Woods & Poole Economics, Inc. Washington, D.C. Copyright 2021. Woods & Poole does not guarantee the accuracy of these data. The use of these data and the conclusions drawn from them are solely the responsibility of the authors of this paper. ^11^ The NHTS data used in this study are from the 2009 NHTS Florida Add-on Program.

**Table 2:** Person-level health outcome models’ estimation results.

	Dependent Variables	Overweight or Obese	Asthma Diagnosis	Diabetes Diagnosis	Good General Health	CDC Physical Activity
Independent Variables						
***Person−Level (Individual and Household) Attributes***						
Age (years)		0.002257 *	0.0058397 ***	0.0045403 ***	−0.004757 ***	−0.0113326 ***
Race (1: white, 0: otherwise)		−0.1855208 ***	NS	−0.0860503 **	0.2192878 ***	0.2454019 ***
Gender (1: male, 0: female)		NS	NS	−0.1197435 ***	NS	0.2160422 ***
Physical activity minutes (per week) ^1^		−0.0105765 ***	−0.0119303 ***	−0.0124975 ***	0.001614 ***	—
Fruits and vegetables (servings per day)		−0.019859 **	−0.0141154 ***	−0.014526 ***	0.0319202 ***	0.0898337 ***
Smoking status (base: nonsmoker)						
everyday smoker		−0.265297 ***	0.1402861 ***	−0.1300801 ***	−0.3827534 ***	NS
someday smoker		−0.240904 ***	0.2390436 ***	−0.1716818 ***	−0.388848 ***	−0.1425182 *
former smoker		NS	0.0975375 ***	NS	−0.2126278 ***	NS
Drinking status (alcoholic beverages per month)		—	—	0.0006407 *	—	—
Household income (base: <$15,000)						
$15,000 to less than $25,000		NS	NS	NS	0.3398008 ***	0.1562742 ***
$25,000 to less than $35,000		NS	NS	NS	0.5259056 ***	0.1643225 ***
$35,000 to less than $50,000		NS	NS	NS	0.7404188 ***	0.2960195 ***
$50,000 or more		NS	−0.2009064 *	NS	1.044106 ***	0.383828 ***
Employment status (1: employed, 0: no) ^2^		NA	NA	NA	0.4421719 ***	NS
College education (1: yes, 0: no) ^2^		NA	NA	NA	0.1382413 ***	0.0963694 **
Children (number of children in household) ^2^		NA	NA	NA	0.0511261 **	NS
***Built Environment Attributes***						
**County Level**						
Mean activity density ^3^		0.0774713 ***	−0.0908846 **	0.0496282 ***	−0.126384 **	0.0915875 **
Mean entropy ^3^		−0.5076122 ***	0.7426236 ***	0.2912284 **	NS	0.5677244 ***
Mean intersection density ^3^		NS	NS	NS	0.3569469 ***	−0.0922825 **
Mean local transit accessibility		NS	−0.0013313 *	NS	−0.0024025 **	0.0026825 ***
Mean temporal automobile accessibility ^3^		NS	0.0509068 *	NS	NS	−0.1099844 **
Mean temporal transit accessibility ^3^		−0.0190041 ***	0.0167732 ***	−0.0121438 ***	NS	NS
Density of fast food restaurants		0.095305 ***	0.1475638 ***	0.1039651 ***	−0.1124666 ***	−0.0771437 ***
Access to parks		−0.0026208 *	0.0056172 ***	−0.002919 ***	NS	0.0088919 ***
Primary care physician rate		−0.0015728 **	NS	NS	0.00129 *	NS
Access to healthy food outlets		−0.0028061 *	−0.0054182 ***	−0.0037962 ***	0.005718 ***	0.0077523 ***
Ambient air pollution		—	0.0040683*	—	−0.0428995 ***	—
Mean Walk Score		−0.0027107 ***	−0.0025144 **	−0.0020266 ***	0.0063055 ***	NS
**Metropolitan Area Level**						
Mean activity density ^3^		NS	−0.2119958 ***	0.1914216 **	NS	NS
Mean entropy ^3^		NS	0.8071199 ***	0.2790995 *	1.445422 ***	1.040943 ***
Mean intersection density ^3^		0.2101968 **	0.1859524*	0.1503409 **	0.3920485 **	NS
Mean local transit accessibility		0.0014559 **	NS	0.000788 **	NS	NS
Mean temporal automobile accessibility ^3^		0.2972497 ***	0.2766411 ***	0.1759324 **	−0.4232195 **	NS
Mean temporal transit accessibility ^3^		NS	0.0411097 ***	−0.0243627 *	0.0784013 ***	0.0830311 ***
Mean roadway congestion index		NS	NS	NS	−0.8122739 **	−0.5009632 *
***Social Environment Attributes***						
**County Level**						
Median age		NS	0.0277286 ***	0.0134414 *	−0.0583641 ***	−0.0476338 ***
Median annual household income		NS	NS	NS	NS	NS
Percentage of white population		NS	NS	NS	0.0261994 ***	0.0127899 ***
Percentage of telecommutable jobs		NS	NS	NS	NS	NS
**Metropolitan Area Level**						
Average percentage of low-wage workers		NS	NS	NS	−0.104597 ***	−0.0800193 ***
Average percentage of households with no cars		NS	NS	NS	−0.1048387 *	NS
Average gross regional product (GRP) ^3^		−0.1058054 **	−0.0756389 *	NS	0.2993636 ***	NS
Average crime rate		0.0003399 ***	−0.0005537 ***	0.000346 ***	−0.000874 ***	−0.0004294 **
***Travel Behavior Attributes***						
**County Level**						
Active travel mode share		−0.0075641 **	NS	NS	0.0054944 *	NS
Private vehicle travel mode share		NS	0.0139775 ***	0.0118146 ***	NS	NS
Public transit travel mode share		−0.0597685 ***	0.0788665 ***	−0.053574 ***	−0.0703956 **	0.0996205 ***
Average frequency of telecommuting events per month		NS	−0.0100291 *	0.0089899 *	−0.0282205 *	−0.0165912 *
Average percentage of household members with telecommuting option		NS	−0.0081331 *	NS	NS	−0.0069587 *
Average number of online purchases per month		0.1019967 **	−0.0466664 *	NS	−0.4156456 ***	NS
Average number of monthly deliveries related to online purchases		NS	NS	0.1015846 **	NS	−0.252159 ***
**Metropolitan Area Level**						
Average walking and bicycling density ^3^		NS	NS	−0.0414568 *	0.1916424 *	NS
Annual public transportation passenger-miles ^3^		NS	0.1228535 ***	−0.0897097 **	−0.2888749 ***	NS
Average commuter stress index		NS	NS	NS	−4.742522 ***	NS
***Other Model Factors***						
Wald test of exogeneity [(corr = 0): χ^2^ (1)] for IV probit model		10.12 ***	11.02 ***	7.49 ***	see Table footer “4”	see Table footer “5”
Amemiya-Lee-Newey minimum χ^2^ test for the equivalent model estimated using the twostep method (test of overidentifying restrictions [61])		0.241 *p*−val. = 0.887	1.619 *p*−val. = 0.445	1.787 *p*−val. = 0.409	NA	NA
Model		IV binary probit	IV binary probit	IV binary probit	Binary probit ^4^	Binary probit ^5^
Log pseudolikelihood		−44672.516	−43961.596	−43214.583	−3026.1687	−4415.5681

^1^ Instrumented variable in IV probit models (on instruments *Employment status*, *College education*, and *Children*). ^2^ Instrumental variable in IV probit models. ^3^ Variable was log-transformed. ^4^ Instrumental variable (IV) analysis showed no endogeneity bias in the model (the Wald test of exogeneity was not significant for the IV probit model—indicating the null hypothesis of no endogeneity cannot be rejected); therefore, a regular binary probit model was estimated instead of an instrumental variable (IV) probit model. ^5^ No endogeneity was assumed in this model; therefore, a regular binary probit model was estimated instead of an instrumental variable (IV) probit model. NA = Not applicable. NS = Not statistically significant.—= Not included in the model. *p*-val. = *p*-value. *, **, *** = Coefficient is significant at the 10%, 5% and 1% significance level, respectively.

**Table 3:** Average marginal effects estimated for the person-level health outcome models.

	Dependent Variables	Overweight or Obese	Asthma Diagnosis	Diabetes Diagnosis	Good General Health	CDC Physical Activity
Independent Variables						
***Person-level (Individual and Household) Attributes***						
Age (years)		NS	0.0016064 ***	0.0031978 ***	−0.0011288 ***	−0.0039874 ***
Race (1: white, 0: otherwise)		−0.0879356 ***	NS	−0.0734162 ***	0.0520407 ***	0.0863445 ***
Gender (1: male, 0: female)		0.168422 ***	−0.0461154 ***	NS	NS	0.0760143 ***
Physical activity minutes (per week) ^1^		−0.0001527*	−0.0000951 **	−0.0001283 **	0.000383 ***	—
Fruits and vegetables (servings per day)		−0.0053932*	NS	NS	0.0075752 ***	0.0316079 ***
Smoking status (base: nonsmoker)						
everyday smoker		−0.0973157 ***	NS	NS	−0.0908339 ***	NS
someday smoker		−0.063421 ***	0.0408043 **	NS	−0.0922802 ***	−0.0501449 *
former smoker		0.0303125*	0.0411301 ***	0.0276464 ***	−0.0504602 ***	NS
Drinking status (alcoholic beverages per month)		—	—	0.0011031 ***	—	—
Household income (base: < $15,000)						
$15,000 to less than $25,000		NS	−0.0672238 ***	NS	0.1129422 ***	0.058242 ***
$25,000 to less than $35,000		NS	−0.0863357 ***	NS	0.1670949 ***	0.061196 ***
$35,000 to less than $50,000		NS	−0.1015195 ***	NS	0.2212181 ***	0.1086166 ***
$50,000 or more		NS	−0.1237543 ***	−0.0652509 ***	0.2818209 ***	0.1390636 ***
Employment status (1: employed, 0: no) ^2^		NA	NA	NA	0.1049349 ***	NS
College education (1: yes, 0: no) ^2^		NA	NA	NA	0.032807 ***	0.0339075 **
Children (number of children in household) ^2^		NA	NA	NA	0.0121331 **	NS
***Built Environment Attributes***						
**County Level**						
Mean activity density ^3^		0.0176133 *	−0.0381007 ***	NS	−0.0299931 **	0.032225 **
Mean entropy ^3^		−0.1795927 *	0.2355872 ***	0.2121117 ***	NS	0.1997535 ***
Mean intersection density ^3^		NS	0.0487475 ***	NS	0.0847095 ***	−0.0324695 **
Mean local transit accessibility		0.0003067 *	−0.0006379 ***	0.0003539 **	−0.0005702 **	0.0009438 ***
Mean temporal automobile accessibility ^3^		0.0360363 *	NS	0.0223294 **	NS	−0.0386979 **
Mean temporal transit accessibility ^3^		−0.0035077 ***	0.0026601 *	NS	NS	NS
Density of fast food restaurants		0.0001297 **	0.0199601 **	0.0131376 *	−0.0266902 ***	−0.027143 ***
Access to parks		NS	0.0019291 ***	−0.0014158 *	NS	0.0031286 ***
Primary care physician rate		−0.0009547 **	NS	NS	0.0003062 *	NS
Access to healthy food outlets		NS	−0.0009925 ***	−0.0012867 *	0.001357 ***	0.0027277 ***
Ambient air pollution		—	0.0020361 *	—	−0.0101808 ***	—
Mean Walk Score		−0.000044 **	−0.0005676 *	NS	0.0014964 ***	NS
**Metropolitan Area Level**						
Mean activity density ^3^		0.1444978 **	NS	NS	NS	NS
Mean entropy ^3^		NS	0.2336963 ***	0.1106682 *	0.3430231 ***	0.3662552 ***
Mean intersection density ^3^		NS	0.0413187 *	0.0443029 *	0.0930397 **	NS
Mean local transit accessibility		0.0003777*	−0.0007915 ***	0.0003052*	NS	NS
Mean temporal automobile accessibility ^3^		NS	NS	NS	−0.1004371 **	NS
Mean temporal transit accessibility ^3^		−0.0213517 **	NS	NS	0.0186059 ***	0.0292144 ***
Mean roadway congestion index		0.2272241 *	0.1200605 **	NS	−0.1927663 **	−0.1762636*
***Social Environment Attributes***						
**County Level**						
Median age		NS	0.0048621 *	NS	−0.0138508 ***	−0.0167599 ***
Median annual household income		NS	−0.00000216 ***	−0.00000224 **	NS	NS
Percentage of white population		NS	0.0020337 *	NS	0.0062176 ***	0.0045001 ***
Percentage of telecommutable jobs		NS	NS	NS	NS	NS
**Metropolitan Area Level**						
Average percentage of low-wage workers		0.0144142 **	NS	NS	−0.0248226 ***	−0.0281547 ***
Average percentage of households with no cars		NS	NS	NS	−0.02488*	NS
Average gross regional product (GRP) ^3^		−0.0442002 **	−0.016817 *	NS	0.071044 ***	NS
Average crime rate		NS	−0.0000583 *	NS	−0.0002074 ***	−0.0001511 **
***Travel Behavior Attributes***						
**County Level**						
Active travel mode share		−0.0015181 **	NS	NS	.0013039*	NS
Private vehicle travel mode share		NS	0.002202 *	NS	NS	NS
Public transit travel mode share		−0.0041469 **	0.0183288 ***	−0.0126945 **	−0.0167061 **	0.0350514 ***
Average frequency of telecommuting events per month		0.0062141 *	−0.0011821 *	NS	−0.0066972 *	−0.0058376 *
Average percentage of household members with telecommuting option		NS	−0.0030147 **	NS	NS	−0.0024484 *
Average number of online purchases per month		0.0633425 **	−0.0261397 *	NS	−0.0986397 ***	NS
Average number of monthly deliveries related to online purchases		0.0714118 **	NS	NS	NS	−0.088722 ***
**Metropolitan Area Level**						
Average walking and bicycling density ^3^		NS	−0.027676 **	−0.0109178 *	0.04548*	NS
Annual public transportation passenger-miles ^3^		−0.0492583 *	NS	−0.0081668 *	−0.0685549 ***	NS
Average commuter stress index		0.7558689 *	NS	NS	−1.125481 ***	NS
Model		IV binary probit	IV binary probit	IV binary probit	Binary probit ^4^	Binary probit ^5^

^1^ Instrumented variable in IV probit models (on instruments *Employment status*, *College education*, and *Children*). ^2^ Instrumental variable in IV probit models. ^3^ Variable was log-transformed. ^4^ Instrumental variable (IV) analysis showed no endogeneity bias in the model (the Wald test of exogeneity was not significant for the IV probit model—indicating the null hypothesis of no endogeneity cannot be rejected); therefore, a regular binary probit model was estimated instead of an instrumental variable (IV) probit model. ^5^ No endogeneity was assumed in this model; therefore, a regular binary probit model was estimated instead of an instrumental variable (IV) probit model. NA = Not applicable. NS = Not statistically significant.—= Not included in the model. *, **, *** = Coefficient (i.e., average marginal effect) is significant at the 10%, 5% and 1% significance level, respectively.

## Data Availability

Publicly available datasets were analyzed in this study. The data sources are listed in the References section along with the relevant URLs, from which, data can be obtained. For the Complete Economic and Demographic Data Source (CEDDS): restrictions apply to the availability of these data. Data were obtained from Woods & Poole Economics, Inc. and can be ordered from their web site at: https://www.woodsandpoole.com/our-databases/united-states/cedds/.
